# Optimum scavenger concentrations for sonochemical nanoparticle synthesis

**DOI:** 10.1038/s41598-023-33243-7

**Published:** 2023-04-15

**Authors:** Henrik E. Hansen, Frode Seland, Svein Sunde, Odne S. Burheim, Bruno G. Pollet

**Affiliations:** 1 Electrochemistry Group, Department of Materials Science and Engineering, Faculty of Natural Sciences, Norwegian University of Science and Technology (NTNU), 7491 Trondheim, Norway; 2 Department of Energy and Process Engineering, Faculty of Engineering, Norwegian University of Science and Technology (NTNU), 7491 Trondheim, Norway; 3 Green Hydrogen Lab, Institute for Hydrogen Research (IHR), Université Du Québec à Trois-Rivières (UQTR), 3351 Boulevard des Forges, Trois-Rivières, Québec G9A 5H7 Canada

**Keywords:** Nanoparticle synthesis, Design, synthesis and processing, Spectrophotometry, Nanoparticles

## Abstract

Maintaining nanoparticle properties when scaling up a chemical synthesis is challenging due to the complex interplay between reducing agents and precursors. A sonochemical synthesis route does not require the addition of reducing agents as they are instead being continuously generated in-situ by ultrasonic cavitation throughout the reactor volume. To optimize the sonochemical synthesis of nanoparticles, understanding the role of radical scavengers is paramount. In this work we demonstrate that optimum scavenger concentrations exist at which the rate of Ag-nanoparticle formation is maximized. Titanyl dosimetry experiments were used in conjunction with Ag-nanoparticle formation rates to determine these optimum scavenger concentrations. It was found that more hydrophobic scavengers require lower optimum concentrations with 1-butanol < 2-propanol < ethanol < methanol < ethylene glycol. However, the optimum concentration is shifted by an order of magnitude towards higher concentrations when pyrolytic decomposition products contribute to the reduction. The reduction rate is also enhanced considerably.

## Introduction

**Figure 1:**
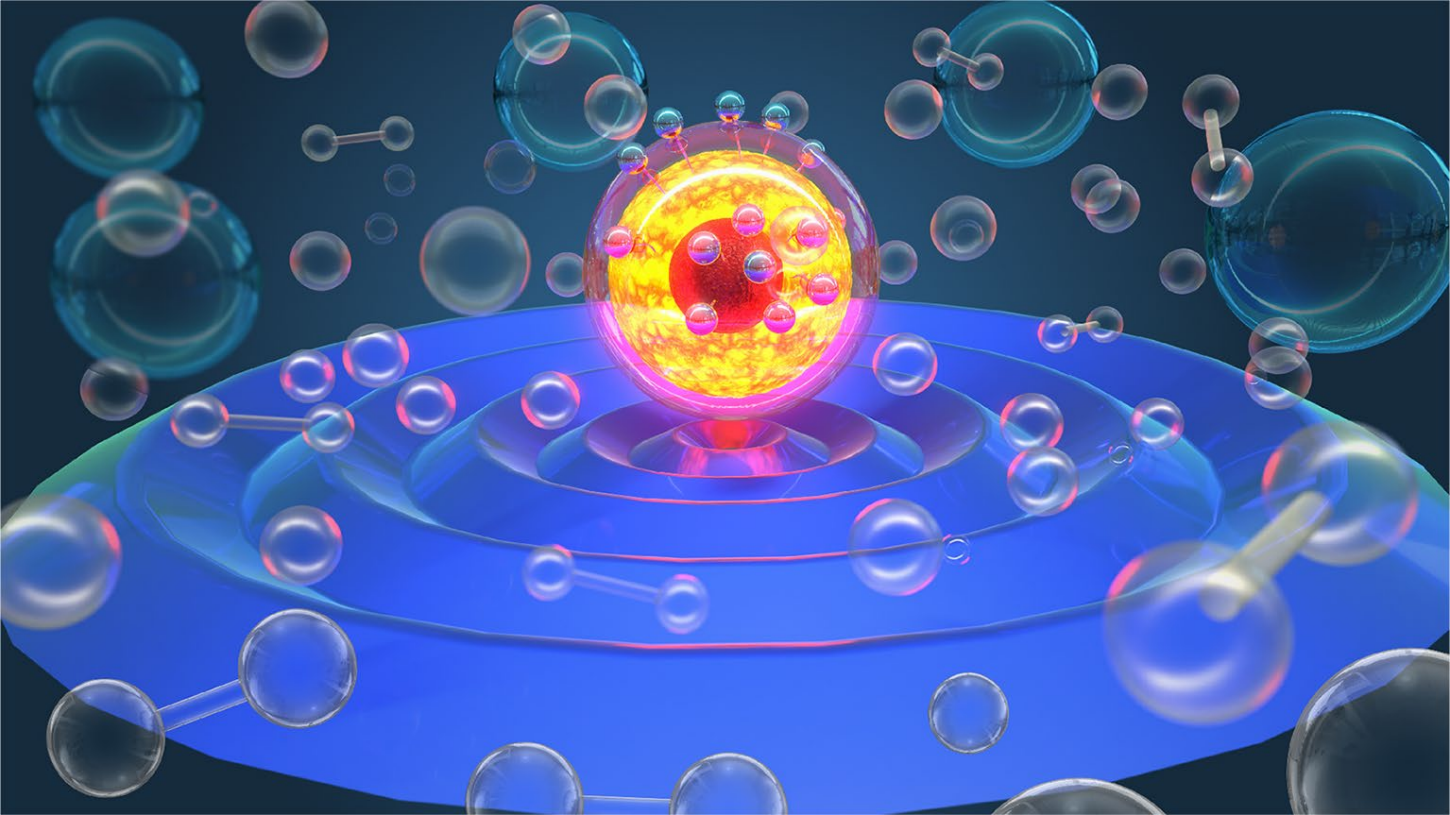
Graphical illustration of the scavenger process in a sonochemical reaction. The central bubble represents the cavitation bubble which achieves extremely high temperatures on collapse. Scavengers are adsorbed on the bubble surface with primary radicals being scavenged by the hydrophobic tail of the scavenger. Incomplete bubble coverage leads to recombination of primary radicals which are illustrated in the surrounding bulk solution. Illustration: George Crystal.

Continued development of many technological applications require the use of the unique properties of nanomaterials. The synthesis methods for these materials must therefore ensure that the desired nanoparticle properties are reproducible. Narrow size distributions, good dispersion, ease of changing the nanoparticle properties as well as the possibility of scaling up the synthesis method are therefore desired traits for a well suited synthesis method. Chemical reduction methods by strong reducing agents such as ${\hbox {NaBH}_{4}}$ are currently being used for nanoparticle synthesis^1–4^. The addition of a strong reducing agent which reacts very quickly with the precursor makes it difficult to obtain a homogeneous mixture. As such different nucleation and growth conditions arise which can result in poor reproducibility. An alternative synthesis method to chemical reduction is the sonochemical synthesis method which utilizes high power ultrasound to generate reducing agents in-situ (Fig. 1).

Sonochemical synthesis of nanoparticles has shown great promise when it comes to tailoring reduction rates and particle sizes^5–12^. This is achieved through adjusting the ultrasonic frequency^7,13^, acoustic power^13^, saturation gas^12–15^, bulk temperature^13,14^, reactor design^16^, and choice of radical scavenger^8,17–19^. Changes in any of these parameters can influence the size of the resulting nanoparticles as they all affect the generation of radicals, which act as reducing agents^7^. However, the sonochemical method is a very slow process as it is dependent upon in-situ generation of primary radicals which is on the order of 10–20 $\upmu \hbox {mol}\,\hbox {dm}^{-3}$ $\hbox {min}^{-1}$ depending on the ultrasound parameters^6,9–12^. If the sonochemical method aims to replace traditional chemical synthesis methods, the utilization of these precious few primary radicals must be optimized. This is ensured by radical scavengers.

The role of a radical scavenger (RH) is to convert the highly unstable primary radicals ($\cdot$H and $\cdot$OH) into more stable reducing secondary radicals ($\cdot$R) as shown through Eq. (2)^6^. Less primary radicals are therefore lost to recombination processes (Eqs. 2–4), and can instead be utilized for reductive purposes (Eq. 5). The choice of a proper scavenger for sonochemical synthesis is very complex as a number of chemical and physical properties must be considered.

1 $$\begin{aligned}{} & {} {\cdot {\text{ OH }}}({\cdot {\text{ H }}}) + {RH} \longrightarrow {R\cdot } + {{\text{ H}}_2{\text{ O }}}({{\text{ H}}_2}) \end{aligned}$$

2 $$\begin{aligned}{} & {} {\cdot {\text{ OH }}} + {\cdot {\text{ H }}} \longrightarrow {{\text{ H}}_2{\text{ O }}} \end{aligned}$$

3 $$\begin{aligned}{} & {} {\cdot {\text{ OH }}} + {\cdot {\text{ OH }}} \longrightarrow {{\text{ H}}_2{\text{ O}}_2} \end{aligned}$$

4 $$\begin{aligned}{} & {} {\cdot {\text{ H }}} + {\cdot {\text{ H }}} \longrightarrow {{\text{ H}}_2} \end{aligned}$$

5 $$\begin{aligned}{} & {} {nR\cdot } + {M^{\mathrm {n+}}} \longrightarrow {nR^{\prime }} + {M} \end{aligned}$$

One such property is the hydrophobicity of the scavenger. To increase the probability of completely scavenging the primary radicals, the radical scavenger should be situated close to the cavitation bubbles^8,20,21^. This can be ensured by choosing a hydrophobic scavenger as it will have a higher affinity towards the gas-solution interface presented by the cavitation bubbles. Such a correlation between scavenging efficiency and hydrophobicity of radical scavengers was demonstrated by Henglein and Kormann for various radical scavengers including glycol, methanol, ethanol, and tert-butanol^20^.

As the hot-spot surrounding a cavitation bubble will cause the scavenger to decompose, the pyrolytic decomposition products of the scavenger must also be considered as these can directly influence the cavitation bubble or the precursor reduction^10,22,23^. Büttner et al.^22^ showed that pyrolytic decomposition of methanol leads to the formation of a methyl radical ($\cdot$CH3) through the pyrolytic cleaving of the C–O bond in methanol. The methyl radical then goes on to form methane, formaldehyde and carbon monoxide through reactions with methanol. Other alcohols, such as ethanol, display similar pyrolytic decomposition reactions as was shown by Gutierrez and Henglein^23^. The introduction of these organic species has been shown to lower the bubble collapse temperature as they evaporate into the cavitation bubbles^17,22^. Fewer primary radicals are therefore produced^14,15,21^.

The methyl radical and other pyrolytic decomposition products have also been shown to contribute in the reduction of certain metal precursor like Pt(IV), Pd(II), and Au(III)^6,10,11^. For these precursors, the rate of reaction is massively increased compared to processes which relies on secondary radicals only. However, not all precursors appear to react with the methyl radical as both the reduction of Ag(I) and Pt(II) to their respective metal nanoparticles only proceed through reactions with secondary radicals^6,9,10,12^.

Even if the scavenging efficiency of the radical scavenger is large, it does not matter if the resulting secondary radical is not able to contribute to the reduction of the metal precursor. For alcohols, hydrogen abstraction from the $\alpha$-carbon leads to the formation of $\alpha$-alcohol radicals which has reducing properties^24^. $\beta$ and higher order alcohol radicals do not have the same reducing properties^24^. Asmus et al.^24^ quantified the yield of hydrogen abstraction from the OH-site, ${\alpha }$-site, and higher order sites for different alcohols and found that the shorter hydrocarbon chains lead to higher yields of ${\alpha }$-alcohol radicals. Both the formate ion and ethylene glycol was shown to exhibit nearly 100% yield of ${\alpha }$-radicals, while n-butanol only displayed a yield of 41.0%.

To achieve faster sonochemical reduction rates, the scavenger must therefore be an efficient primary radical scavenger, and the resulting secondary radical must be able to readily transfer electrons to the metal precursor. Ideally, the pyrolytic decomposition products should also contribute to the reduction before they are converted into volatile gases. However, if they are inactive towards the reduction, their concentration should be minimized. A higher scavenging efficiency will therefore increase sonochemical reduction, while excessive pyrolytic decomposition will decrease the sonochemical reduction. As the scavenging efficiency and pyrolytic decomposition both increase with scavenger concentration, an optimum scavenger concentration for faster sonochemical reduction should therefore exist.

In this work, the optimum scavenger concentrations for methanol, ethanol, 1-butanol, 2-propanol, and ethylene glycol were determined for the sonochemical reduction and formation of Ag-nanoparticles. This was performed through identifying the lowest alcohol concentrations required to reach complete scavenging of primary radicals through absorbance-based detection of ${{\hbox {H}}_2{\hbox {O}}_2}$. ${{\hbox {H}}_2{\hbox {O}}_2}$ detection offers useful insight into the degree of scavenging as it will form by recombination of primary radicals in the absence of a scavenger. These results were then compared to the formation rates of Ag-nanoparticles under the same conditions. To determine whether pyrolytic decomposition products affect the optimum scavenger concentration or not, the sonochemical reduction of Pt(IV) to Pt(II) was also assessed for different methanol concentrations. The effect of acoustic power and ultrasonic frequency on the scavenging efficiency was also considered in order to determine the extent to which these must be taken into account when optimizing the scavenger concentrations.

## Results

**Figure 2:**
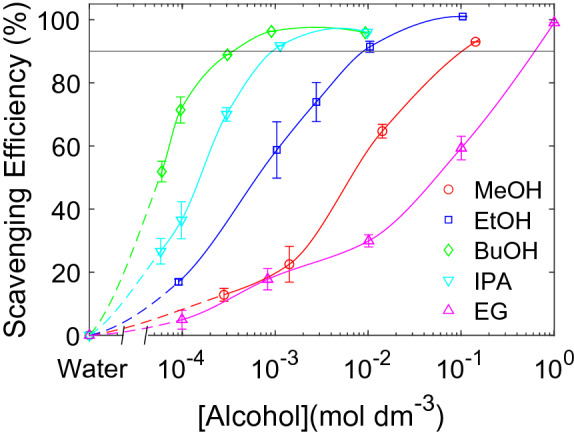
Scavenging efficiency of $\cdot$OH radicals as a function of alcohol concentration. Methanol (MeOH) ($\bigcirc$), ethanol (EtOH) ($\Box$), 1-butanol (BuOH) ($\Diamond$), 2-propanol (IPA) ($\triangledown$), and ethylene glycol (EG) ($\triangle$) were used as radical scavengers. Solid and dotted lines are drawn with spline interpolation to guide the eye. The horizontal line at 90% represents the upper detection limit for the scavenging efficiency.

The scavenging efficiency of $\cdot$OH radicals for different alcohols and alcohol concentrations are shown in Fig. 2. Scavenging efficiency is a measure of how much primary radicals are converted into secondary radicals through scavenging. The alcohol concentration required to reach the scavenging efficiency limit (90%) is lower for the more hydrophobic scavengers. These concentrations are approximately 0.3 ${\hbox {mmol}\,\hbox {dm}^{-3}}$ for 1-butanol, 1 ${\hbox {mmol}\,\hbox {dm}^{-3}}$ for 2-propanol, 10 ${\hbox {mmol}\,\hbox {dm}^{-3}}$ for ethanol, 100 ${\hbox {mmol}\,\hbox {dm}^{-3}}$ for methanol, and 500 ${\hbox {mmol}\,\hbox {dm}^{-3}}$ for ethylene glycol. Absorbance spectra and the corresponding concentration profiles (Figs. S1–S5), and ${{\hbox {H}}_2{\hbox {O}}_2}$ rates as a function of alcohol concentration (Fig. S6) for all samples are provided in the Supporting information.

**Figure 3:**
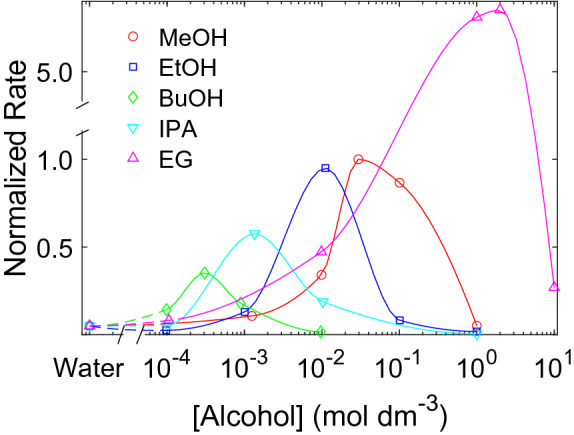
Rate of Ag-nanoparticle formation as a function of alcohol concentration. Methanol (MeOH) ($\bigcirc$), ethanol (EtOH) ($\Box$), 1-butanol (BuOH) ($\Diamond$), 2-propanol (IPA) ($\triangledown$), and ethylene glycol (EG) ($\triangle$) were used as radical scavengers. The rate of Ag-nanoparticle formation is normalized to the optimum for the methanol series. Solid and dotted lines are drawn with spline interpolation to guide the eye.

The sonochemical formation rates of Ag-nanoparticles in the presence of different alcohols and alcohol concentrations are shown in Fig. 3. The rates are normalized to the maximum formation rate in the methanol series and are corrected for shifts in the localized surface plasmon resonance (LSPR) peak. All alcohols exhibit a maximum rate of Ag-nanoparticle formation. The alcohol concentration required to reach maximum Ag-nanoparticle formation is lower for the more hydrophobic scavengers. These concentrations are approximately 0.3 ${\hbox {mmol}\,\hbox {dm}^{-3}}$ for 1-butanol, 1 ${\hbox {mmol}\,\hbox {dm}^{-3}}$ for 2-propanol, 10 ${\hbox {mmol}\,\hbox {dm}^{-3}}$ for ethanol, 30 ${\hbox {mmol}\,\hbox {dm}^{-3}}$ for methanol and 2 ${\hbox {mol}\,\hbox {dm}^{-3}}$ for ethylene glycol. The maximum rate for ethylene glycol was found to be 5.4 times higher than the maximum rate for methanol. Absorbance spectra and the corresponding concentration profiles (Figs. S7–S11) for all Ag-samples are provided in the Supporting information. Ag-nanoparticle formation rates without LSPR peak correction are also provided in the Supporting information (Fig. S12). Nitrate ions have previously been shown to affect the scavenging of OH radicals^25,26^. However, the nitrate ions present in ${\hbox {AgNO}_{3}}$ in our work have a negligible effect towards the scavenging efficiency as demonstrated in Fig. S13 in the Supporting information. Representative SEM micrographs of the resulting Ag-nanoparticles are also provided in the Supporting information (Fig. S14a) along with the corresponding EDS-map of Ag (Fig. S14b).

**Figure 4:**
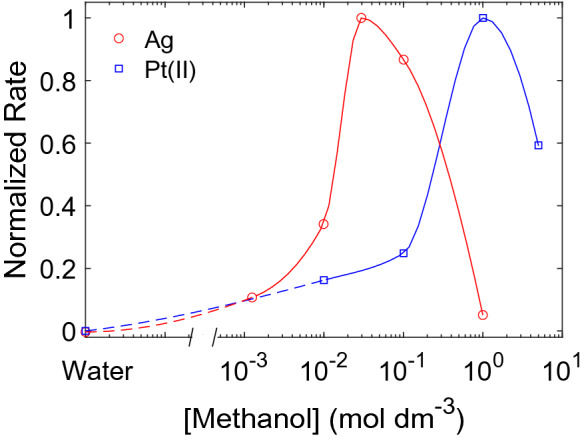
Normalized rate of Ag-nanoparticle formation ($\bigcirc$) and Pt(II) formation ($\Box$) as a function of methanol concentration. Solid and dotted lines are drawn with spline interpolation to guide the eye.

Normalized sonochemical formation rates of Ag-nanoparticles and Pt(II) at different methanol concentrations are shown in Fig. 4. The methanol concentration required to reach maximum Pt(II) formation $({1}\,{\hbox {mol}\,\hbox {dm}^{-3}})$ is an order of magnitude higher than what is needed to reach maximum Ag-nanoparticle formation $({30}\,{\hbox {mmol}\,\hbox {dm}^{-3}})$. Absorbance spectra and the corresponding concentration profiles of Pt(II) (Fig. S15), and the non-normalized Pt(II) rates (Fig. S16) are provided in the Supporting information.

**Figure 5:**
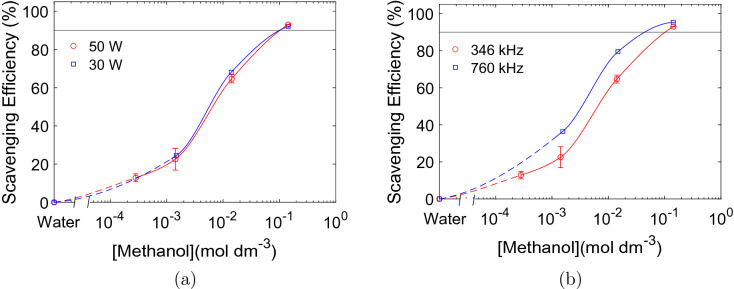
Scavenging efficiency of $\cdot$OH radicals as a function of methanol concentration for applied electrical powers of 50 W ($\bigcirc$) and 30 W ($\Box$) (**a**), and for applied ultrasonic frequencies of 346 kHz ($\bigcirc$) and 760 kHz ($\Box$) (**b**). The power experiments in (**a**) were acquired at an applied ultrasonic frequency of 346 kHz, while the frequency experiments in (**b**) were acquired at an applied electrical power of 50 W. Solid and dotted lines are drawn with spline interpolation to guide the eye. The horizontal lines at 90% represents the upper detection limit for the scavenging efficiency.

The effect of acoustic power and ultrasonic frequency on the methanol scavenging efficiency is shown in Fig. 5a,b, respectively. No significant differences can be observed between high and low ultrasonic powers, and they both reach the scavenging efficiency limit (90%) at the same methanol concentration $({100}\,{\hbox {mmol}\,\hbox {dm}^{-3}})$. However, when the ultrasonic frequency is increased from 346 to 760 kHz the scavenging efficiency does increase. Complete scavenging of $\cdot$OH radicals therefore appears to occur at lower methanol concentrations at 760 kHz $({50}\,{\hbox {mmol}\,\hbox {dm}^{-3}})$ compared to 346 kHz $({100}\,{\hbox {mmol}\,\hbox {dm}^{-3}})$. Absorbance spectra and the corresponding concentration profiles for measurements conducted at 30 W (Fig. S17) and 760 kHz (Fig. S18) are provided in the Supporting information.

## Discussion

The optimal scavenger concentration for sonochemical formation of Ag-nanoparticles is achieved for the concentration at which the scavenger exactly covers the cavitation bubble. This is supported by the good agreement between the alcohol concentration required for complete radical scavenging (Fig. 2) and maximum Ag-nanoparticle formation rate (Fig. 3). At lower alcohol concentrations, primary radicals are lost to recombination due to insufficient bubble coverage. At higher alcohol concentrations pyrolytic decomposition of excess alcohol contributes to a lower formation rate of primary radicals. This is in line with the decrease in Ag-nanoparticle formation rates at low and high alcohol concentrations (Fig. 3).

If the cavitation bubble is not completely covered by the scavenger, the primary radicals may recombine in scavenger-deficient areas of the hot-spot^27^. This is consistent with the low scavenging efficiencies observed at low alcohol concentrations (Fig. 2). Low scavenging efficiencies means that less secondary radicals are formed. Correspondingly low rates are therefore observed for the Ag-nanoparticle formation (Fig. 3) because it proceeds through reactions with secondary radicals^12^. As the alcohol concentration increases, more of the cavitation bubbles are covered by the scavenger and less recombination occurs. This is evident from the high scavenging efficiencies observed at higher scavenger concentrations (Fig. 2). As a result, more secondary radicals are generated which increases the Ag-nanoparticle formation rates (Fig. 3).

Increasing the scavenger concentration beyond complete bubble coverage does not contribute to more scavenging of primary radicals. However, the excess scavenger will still be subject to the high temperatures that are associated with the hot-spot region^28^. Previous studies on decomposition of alcohols through high frequency ultrasound have shown that alkanes and alkenes are formed as gaseous decomposition products^22,23^. Accumulation of these compounds in the cavitation bubbles will alter the collapse conditions of the bubbles. This is because the polytropic ratio ($\gamma$) is pushed towards lower values when moving from monoatomic Ar (1.66) to polyatomic organic species (1.3–1.2)^17,21,29^. A decrease in the polytropic ratio leads to a decrease in the bubble collapse temperature (*T*)

6 $$\begin{aligned} T = T_{\rm{bulk}}\left( \frac{R_{\rm{max}}}{R_{0}}\right) ^{3(\gamma - 1)} \end{aligned}$$

where $T_{\rm{bulk}}$ is the bulk temperature of the solution, $R_{\rm{max}}$ is the maximum radius of the cavitation bubble and $R_0$ is the ambient radius of the cavitation bubble^14^. A lower collapse temperature also leads to fewer primary radicals being formed^14^. As a result, the rate of secondary radical formation is also reduced. The slower Ag-nanoparticle formation rate at high alcohol concentrations can therefore be attributed to pyrolytic decomposition of the excess scavenger.

To achieve maximum Ag-nanoparticle formation rates, secondary radical formation must therefore be optimized. Secondary radical formation is maximized when the scavenger concentration is high enough to scavenge all primary radicals, but low enough to limit pyrolytic decomposition of the scavenger. This occurs once the scavenger exactly covers the cavitation bubbles. The observed maximum in Ag-nanoparticle formation rates (Fig. 3) does indeed coincide with the concentration where complete bubble coverage is reached (Fig. 2). We therefore conclude that maximum Ag-nanoparticle formation rates can be achieved for scavenger concentrations at which complete bubble coverage occurs.

Maximum Ag-nanoparticle formation occurring at complete bubble coverage is also supported when comparing the shift in optimum scavenger concentrations between different alcohols (Fig. 3). The trend follows the hydrophobicity of the scavengers with the most hydrophobic scavenger (1-butanol) displaying the lowest optimum concentration, and the least hydrophobic scavenger (ethylene glycol) displaying the highest optimum concentration (1-butanol < 2-propanol < ethanol < methanol < ethylene glycol). This is the same trend we observe for the scavenging efficiency experiments (Fig. 2). It is also the same trend as Henglein and Kormann^20^ observed for their scavenging efficiency experiments which they attributed to the higher degree of bubble coverage displayed by the more hydrophobic scavengers. This further strengthens our finding that the Ag-nanoparticle formation rate is determined by the degree of bubble coverage.

In addition to a shift in optimum scavenger concentration, the relative rates of Ag-nanoparticle formation also appear to be scavenger dependent. The relative rates of Ag-nanoparticle formation for the different scavengers can be related to the conversion efficiency from primary radicals to $\alpha$-alcohol secondary radicals, where the shorter hydrocarbon chains display higher conversion efficiencies. This is shown from the trends in the normalized rates (Fig. 3).

When an alcohol scavenger interacts with a primary radical, the radical may abstract a hydrogen atom from any position in the alcohol^24^. The longer the chain length the lower is the probability of hydrogen abstraction from the $\alpha$-site which is the only site capable of proper electron transfer to a metal precursor^24^. Abstraction probabilities from the $\alpha$-site were estimated for methanol (93.0%), ethanol (84.3%), 2-propanol (85.5%), and n-butanol (41%) by Asmus et al.^24^. The relative rates we observe for the Ag-nanoparticle formation rates (Fig. 3) appear to be consistent with the abstraction probabilities of the linear alcohols.

For ethylene glycol, the relative rate of Ag-nanoparticle formation was observed to be about five times higher than for methanol. It also reached a maximum rate for ethylene glycol concentrations one order of magnitude higher than what is needed for complete bubble coverage (Fig. 3). A fast rate of Ag-nanoparticle formation is indeed expected when using ethylene glycol as approximately 100% of the secondary radicals are $\alpha$-alcohol secondary radicals^24^. However, the extremely fast rate and an optimum scavenger concentration which is higher than expected suggests that there are other factors contributing to the reduction process. It is well known that ethylene glycol is used extensively in polyol-reduction methods for synthesizing nanoparticles^30,31^. This occurs at elevated temperatures where ethylene glycol decomposes into species with reducing capabilities. Excess ethylene glycol in the hot-spot region could therefore decompose from the high temperatures, but instead of negatively affecting the bubble collapse like the other alcohols, the decomposition products could act as additional reducing agents. This might explain why the relative rate of Ag-nanoparticle formation is so much higher than for methanol, and also why the optimum scavenger concentration is shifted to higher ethylene glycol concentrations.

To assess the extent to which the Ag-nanoparticle formation is aided by pyrolytic decomposition of ethylene glycol, the reduction of Pt(IV) to Pt(II) was monitored for different methanol concentrations (Fig. 4). The reduction of Pt(IV) to Pt(II) has also been shown to proceed through pyrolytic decomposition products^10^. This process is driven by the decomposition products from alcohols in general. We observe that the Pt(II) formation rate also displays a maximum for methanol concentrations one order of magnitude higher than the Ag-nanoparticle formation (Fig. 4). This is very similar to what we observe for ethylene glycol. The shift in the optimum scavenger concentration for Pt(II) may therefore be attributed to the contribution from the decomposition products towards the reduction. Going beyond complete bubble coverage would therefore increase the number of reducing agents which would lead to further increase in the rate of nanoparticle formation. However, lower collapse temperatures associated with these decomposition products will eventually start to dominate at higher scavenger concentrations leading to lower reduction rates. We therefore conclude that the optimum scavenger concentration is shifted towards higher concentrations when the decomposition products actively participates in the sonochemical reduction.

Thus far we have shown that the scavenger concentration and hydrophobicity determine how much of the bubble area that is covered by the scavenger. If the bubble area is modified through changes in the ultrasound parameters, the bubble coverage might change as well. As a result, we would need to find optimum scavenger concentrations for a given set of ultrasound parameters as well.

An increase in ultrasonic power above 10 W increases the number of cavitation bubbles, but the size of each cavitation bubble remains fairly constant^32^. This leads to a larger total surface area, but the individual bubble area is unchanged. The scavenging efficiency was found to be independent on the ultrasonic power (Fig. 5a). The bubble coverage is therefore independent on the number of cavitation bubbles.

Increasing the ultrasonic frequency has several effects on the cavitation bubbles. It reduces the size of each bubble, increases the number of cavitation bubbles, reduces the collapse times, decreases the final shock wave pressure, and decreases the collapse temperatures and therefore also the amount of primary radicals generated per bubble. The scavenging efficiencies of methanol at 346 kHz and 760 kHz (Fig. 5b) reveals that an increase in frequency increases the bubble coverage for the same methanol concentration. From the power measurements, we know that the bubble coverage is independent of the number of cavitation bubbles. The decrease in the single bubble surface area may affect the bubble coverage through changes in diffusion of the alcohol towards the bubble surface. However, the diffusion process may also be affected by the different collapse times, shock wave pressures and collapse temperatures imposed by the ultrasonic frequency. When optimizing the scavenger concentration, the ultrasonic frequency must therefore be taken into account. An optimum concentration at high ultrasonic frequencies would therefore not be sufficient at lower frequencies which would require a higher scavenger concentration.

## Conclusions

For sonochemical reduction of metal precursors to nanoparticles, optimum scavenger concentrations do exist. This depends on the hydrophobicity and pyrolytic decomposition products of the scavenger. More hydrophobic scavengers reduce the optimum scavenger concentration due to their higher affinity towards the bubble solution interface. If the pyrolytic decomposition products of the scavenger are active towards the precursor reduction, the optimum scavenger concentration and the rate of reduction increases. For scavengers without active pyrolytic decomposition products, the optimum scavenger concentration coincides with the scavenger concentration required to reach complete bubble coverage. This is simply determined as the lowest scavenger concentration which yields undetectable amounts of ${{\hbox {H}}_2{\hbox {O}}_2}$. It was also found that the optimum scavenger concentrations are dependent on the ultrasonic frequency and independent on the ultrasonic power for the acoustic powers and frequencies used in this work.

## Methods

### Chemicals

Methanol (MeOH) (Acros Organics, 99.8% for electronic use), ethanol (EtOH) (VWR, 96% GPR RECTAPUR ®), 1-butanol (BuOH) (Merck, $\ge$ 99.5% EMSURE®ACS, ISO, Reag. Ph Eur), 2-propanol (IPA) (VWR, 98% technical), ethylene glycol (EG) (Sigma Aldrich, $\ge$ 99.0% ReagentPlus®), sodium nitrate $({\hbox {NaNO}_{3}}$)(Merck, EMSURE®ACS, ISO, Reag. Ph Eur), silver nitrate $({\hbox {AgNO}_{3}})$ (Sigma Aldrich, $\ge$ 99.0% ACS reagent), platinum tetrachloride $({\hbox {PtCl}_{4}})$ (Sigma Aldrich, 96%), titanium(IV) oxysulfate solution $({\hbox {TiOSO}_{4}})$ (Sigma Aldrich, 1.9- 2.1%), and potassium iodide (KI) (Sigma Aldrich, $\ge$ 99% ACS reagent) were used as received from the supplier. Milli-Q water $({18.2} M\Omega \cdot \hbox {cm})$ was used for all experiments.

#### Sonochemical setup

All sonochemical experiments were performed using Honda Electronics 70 mm $\varnothing$ stainless steel alloy (SUS304) plates connected to lead zirconate titanate (PZT) piezoelectric transducers (346 kHz and 760 kHz). Temperature control (3 $^{\circ }\hbox {C}$) was maintained through water circulation around the reactor. Atmospheric gasses where removed from solution through constant supply of argon (5.0) before and during the sonochemical experiments. An AG 1012 RF signal generator from T &C Power Conversion was used in combination with the plate transducers to generate the ultrasonic waves. Proper transfer of the power from the signal generator to the transducer was ensured through an impedance matching unit (T1k-7A) from T &C Power Conversion. In our previous work, the conversion efficiency from electric power (50 W) to acoustic power (38(3)W) was found to be 76(6)%^33^. Detailed schematics of the sonochemical reactor can be found in our previous work^34^.

#### Alcohol scavenging efficiency

The scavenging properties of methanol, ethanol, 2-propanol, 1-butanol, and ethylene glycol were determined through titanyl dosimetry. 200mL aqueous solutions with scavenger concentrations between 60 $\upmu \hbox {mol}\,{\hbox {dm}}^{-3}$ and 1 $\hbox {mol}\,{\hbox {dm}}^{-3}$ were sonicated for 20 min at 346 kHz and 50 W electric power. 500 $\upmu \hbox {L}$ aliquots were taken every 5 min and mixed with 500 $\upmu \hbox {L}$ of 0.02 $\hbox {mol}\,\hbox {dm}^{-3}$ ${\hbox {TiOSO}_4}$^18,26,35,36^. The absorbance spectra of these solutions were acquired with a scan rate of 100 ${\hbox {nm}\,\hbox {min}^{-1}}$ between 300 and 600 nm with an Evolution 220 UV–visible spectrophotometer from Thermo Fisher. The ${\hbox {H}_2\hbox {O}_2}$ concentration for all samples was determined from the absorbance peak at 411 nm ($\epsilon$ = 787  $\hbox {mol}^{-1}\,\hbox {dm}^{3}\,\hbox {cm}^{-1}$) belonging to the yellow titanium hydrogen peroxide complex^34^. The ${\hbox {H}_2\hbox {O}_2}$ formation rates at the given alcohol concentrations were determined from the slopes of the resulting linear concentration profiles. The rates were then converted to scavenging efficiencies (*S*) by comparing the rate of ${{\hbox {H}}_{2}{\hbox {O}}_{2}}$ production with a scavenger present $(r_{\hbox {S}})$ and without a scavenger present $(r_{0}={5.5}\,\upmu \hbox {mol}\,{\hbox {dm}}^{-3}\,{\hbox {min}}^{-1})$.

7 $$\begin{aligned} S = \left( 1 - \frac{r_{\rm{S}}}{r_{\rm{0}}}\right) \times 100\% \end{aligned}$$

A lower detection limit for ${{\hbox {H}}_2{\hbox {O}}_2}$ of 10 ${\hbox {mumol}\,{\hbox {dm}}^{-3}}$ after 20 min sonication was used as an indicator of complete scavenging for the different alcohols. This was identified as the ${{\hbox {H}}_2{\hbox {O}}_2}$ concentration where the absorbance peak at 411 nm was no longer discernible from the background. From Eq. (7), a scavenging efficiency above 90% can therefore be regarded as complete scavenging of the primary radicals. The alcohol concentrations were increased until complete scavenging was achieved. Three replicate experiments were performed for all scavenger concentrations below the scavenging efficiency limit. The standard deviation from these replicates were then used as error bars in the resulting graphs. The scavenging efficiency of methanol was also determined at 760 kHz with a 50 W electric power, and at 346 kHz with a 30 W electric power to assess the effect of ultrasonic frequency and power.

#### Sonochemical reduction rate of silver nitrate

The reducing capabilities of the secondary radicals were evaluated in terms of the sonochemical reduction of Ag(I) to Ag-nanoparticles. The Ag(I) reduction was chosen specifically because it has been shown to proceed mainly through reactions with secondary radicals^6,12^. 200 mL of 10 $\,\hbox {mmol dm}^{-3}$ $\hbox {AgNO}_{3}$ solutions with scavenger concentrations between 100 $\upmu \hbox {mol}\,\hbox {dm}^{-3}$ and 10  $\hbox {mol}\,\hbox {dm}^{-3}$ were sonicated for 20 min at 346 kHz and 50W electric power. 500 $\upmu \hbox {L}$ aliquots were taken every 5 min and mixed with 500 $\upmu \hbox {L}$ of Milli-Q water. The absorbance spectra of these solutions were acquired with a scan rate of 100 nm min$^{-1}$ between 200nm and 800nm with an Evolution 220 UV–visible spectrophotometer from Thermo Fisher. Ag-nanoparticle formation rates were estimated from the development of the localized surface plasmon resonance (LSPR) peak in the absorbance spectra which is located between 400 and 500nm.

As the exact wavelength of the LSPR peak of Ag-nanoparticles is dependent on the Ag-nanoparticle size, nanoparticle growth throughout the sonication period will shift the LSPR peak to higher wavelengths and therefore result in different molar extinction coefficients for the Ag-nanoparticles. In order to compensate for the LSPR peak shift throughout the sonication period and between different samples, the Ag-nanoparticle concentration had to be evaluated with the molar extinction coefficient corresponding to the individual LSPR peak positions. In a work by Paramelle et al.^37^ they measured the molar extinction coefficient of citrate capped Ag-nanoparticles for LSPR peak positions between 392.1 and 492.8 nm. They found that the molar extinction coefficient is proportional to the square of the LSPR peak position. From their data we corrected the Ag-nanoparticle absorbance values at every sample interval by matching the observed LSPR peak positions with the corresponding molar extinction coefficients. The calibration curve relating the molar extinction coefficient to the peak wavelength is provided in the Supporting information (Fig. S19). To estimate the rate of Ag-nanoparticle formation for the different scavengers, the slope of the absorbance profiles at 0 min was used.

#### Sonochemical reduction rate of platinum chloride

The reduction of ${\hbox {PtCl}_4}$ from Pt(IV) to Pt(II) was chosen to investigate the scavenger concentration dependence when pyrolytic decomposition also plays a part in the reduction process. 200 mL of 1 ${\hbox {mmol}\,\hbox {dm}^{-3}}$ ${\hbox {PtCl}_4}$ solutions with methanol concentrations between 10 ${\hbox {mmol}\,\hbox {dm}^{-3}}$ and 5 ${\hbox {mol}\, \hbox {dm}^{-3}}$ were sonicated for 20 min at 346 kHz and 50 W electric power. 100 $\upmu \hbox {L}$ aliquots were taken every 5 min and mixed with 100 $\upmu {\hbox {L}}$ of KI and 800 $\upmu \hbox {L}$ of Milli-Q water. The resulting ${\hbox {PtI}_{6}^{2-}}$ ($\epsilon$ = 11,170 $\hbox {dm}^{3}\, \,\hbox {mol}^{-1} \,\hbox {cm}^{-1}$, $\lambda _{\rm{max}}$ = 495 nm) and ${\hbox {PtI}_{4}^{2-}}$ ($\epsilon$ = 4600$\,\hbox {dm}^{3}\, \,\hbox {mol}^{-1} \,\hbox {cm}^{-1}$, $\lambda _{\rm{max}}$ = 388 nm) complexes were determined spectrophotometrically as described in our previous work^34^. The formation rates of Pt(II) for the different methanol concentrations were determined as the slope of the Pt(II) concentration profiles at 0 min.

## Supplementary Information

Supplementary Information.

## Data Availability

The datasets used during the current study are available from the corresponding author on reasonable request.
