# Supplementary material for: Optimum scavenger concentrations for sonochemical nanoparticle synthesis
Source: Sci Rep. 2023 Apr 15;13:6183. doi: 10.1038/s41598-023-33243-7 (PMC10105774; doi:10.1038/s41598-023-33243-7)
Supplement: Supplementary file 1 — Supplementary Information. [file 41598_2023_33243_MOESM1_ESM.pdf]

Supporting Information

Titanyl Dosimetry Results

Absorbance spectra showing the development of H_2O_2 for different sonication times along with their corresponding concentration profiles are given in Figure S1, S2, S3, S4, and S5 for methanol, ethanol, 1-butanol, 2-propanol, and ethylene glycol, respectively. Rates of H_2O_2 formation in the presence of different alcohols and alcohol concentrations are shown in Figure S6.

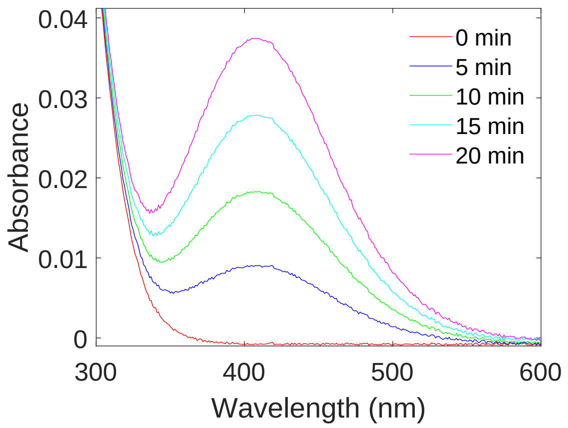

(a)

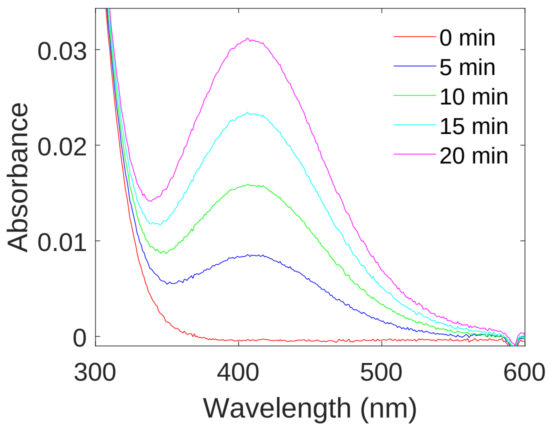

(b)

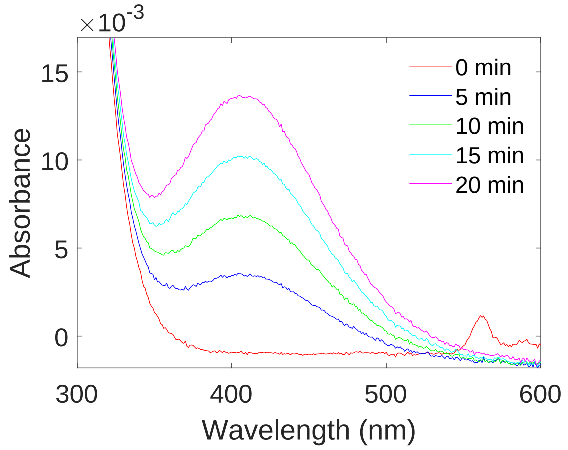

(c)

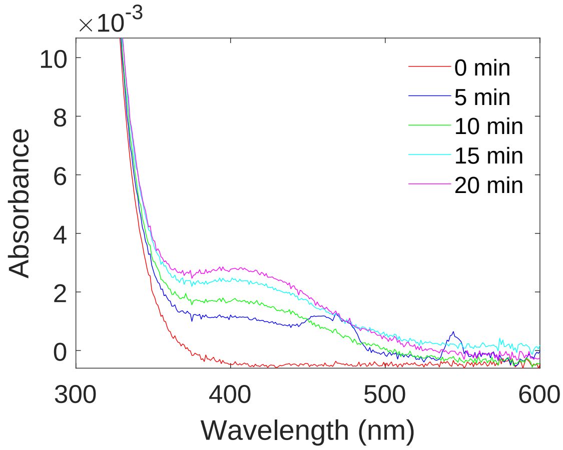

(d)

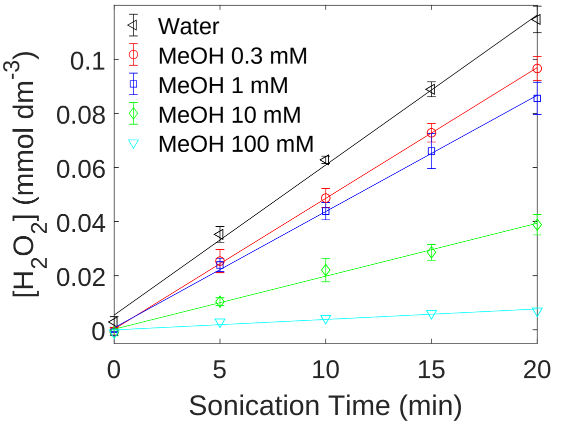

(e)

Figure S1: Absorbance spectra of titanium hydrogen peroxide complexes at different sonication times acquired using methanol as the radical scavenger. Initial methanol concentrations of 0.3 mmol dm^{-3} S1a, 1.0 mmol dm^{-3} S1b, 10 mmol dm^{-3} S1c, and 100 mmol dm^{-3} S1d were used. The resulting H_2O_2 concentrations for all methanol (MeOH) concentrations are also plotted as a function of sonication time S1e.

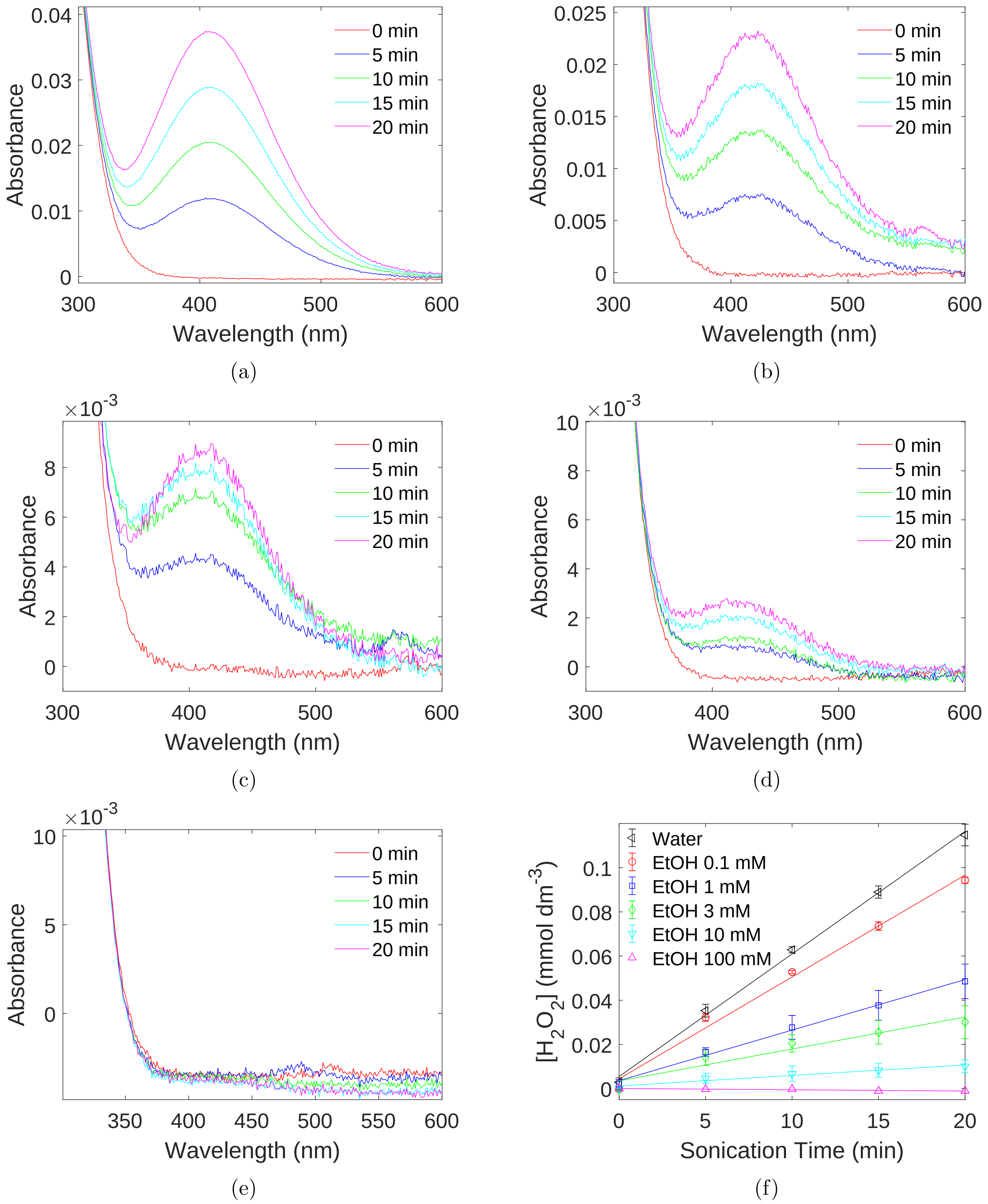

Figure S2: Absorbance spectra of titanium hydrogen peroxide complexes at different sonication times acquired using ethanol as the radical scavenger. Initial ethanol concentrations of 0.1 mmol dm^{-3} S2a, 1.0 mmol dm^{-3} S2b, 3.0 mmol dm^{-3} S2c, 10 mmol dm^{-3} S2d, and 100 mmol dm^{-3} S2e were used. The resulting H_2O_2 concentrations for all ethanol (EtOH) concentrations are also plotted as a function of sonication time S2f.

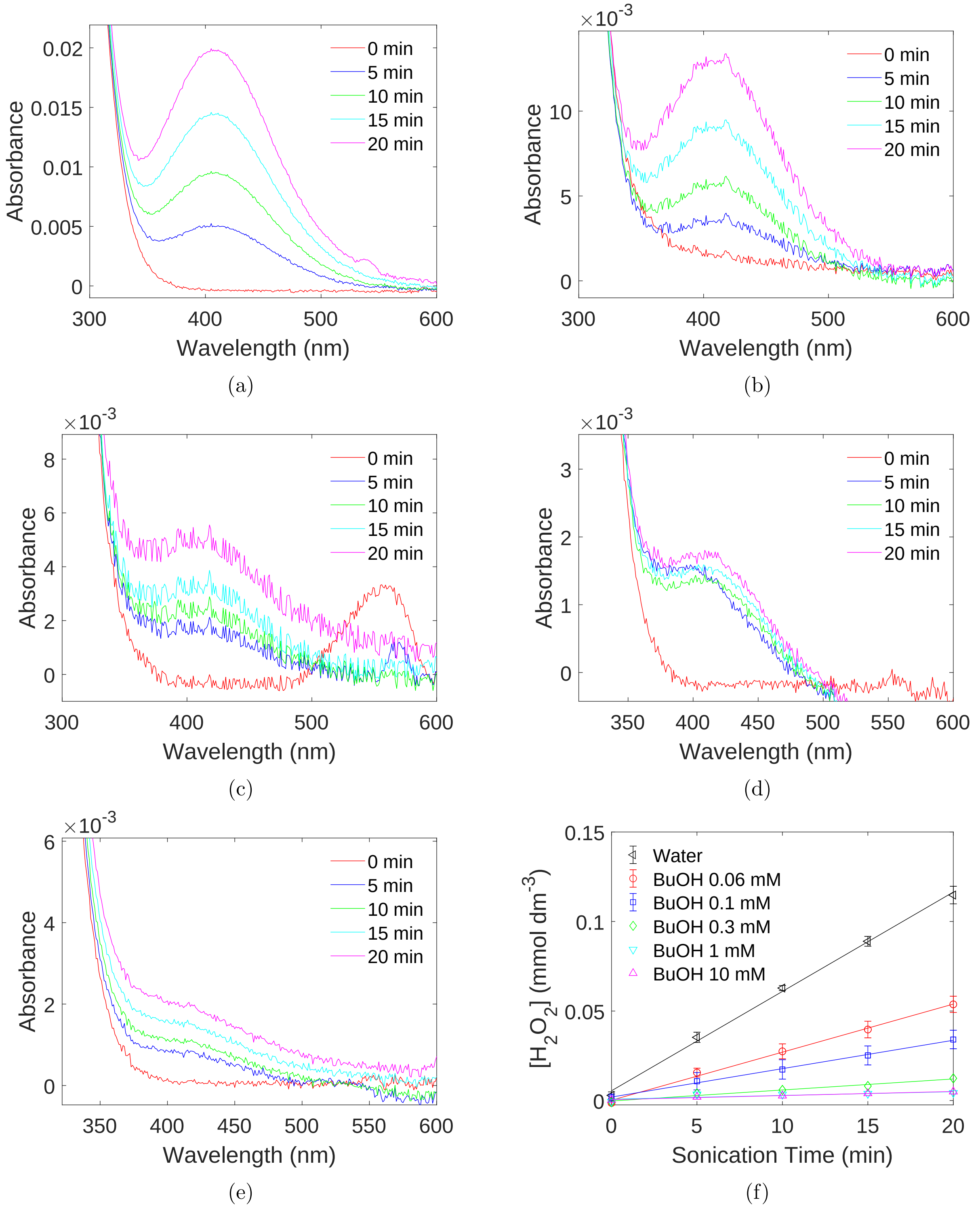

Figure S3: Absorbance spectra of titanium hydrogen peroxide complexes at different sonication times acquired using 1-butanol as the radical scavenger. Initial 1-butanol concentrations of 0.06 mmol dm^{-3} S3a, 0.1 mmol dm^{-3} S3b, 0.3 mmol dm^{-3} S3c, 1.0 mmol dm^{-3} S3d, and 10 mmol dm^{-3} S3e were used. The resulting H_2O_2 concentrations for all 1-butanol (BuOH) concentrations are also plotted as a function of sonication time S3f.

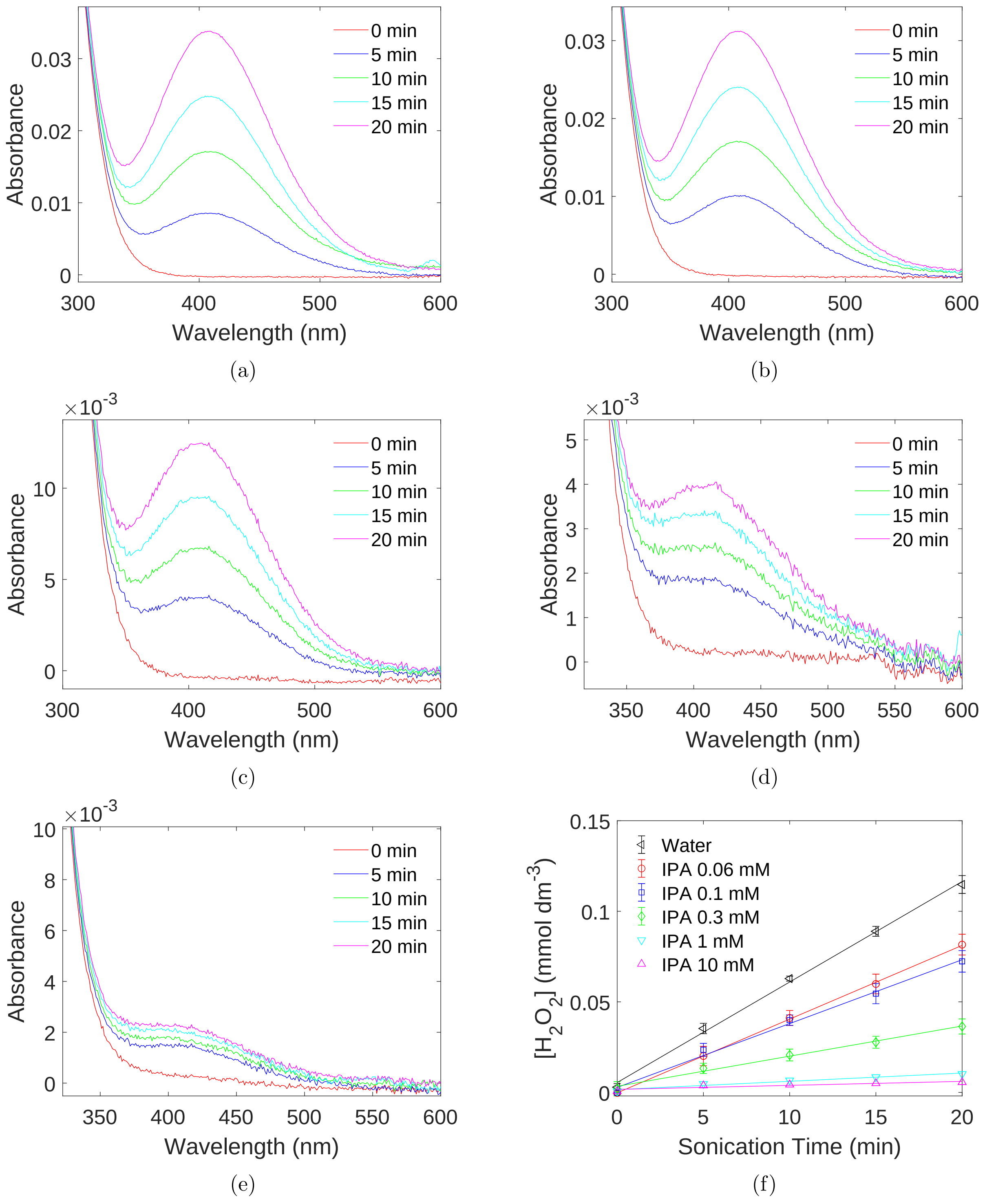

Figure S4: Absorbance spectra of titanium hydrogen peroxide complexes at different sonication times acquired using 2-propanol as the radical scavenger. Initial 2-propanol concentrations of 0.06 mmol dm^{-3} S4a, 0.1 mmol dm^{-3} S4b, 0.3 mmol dm^{-3} S4c, 1.0 mmol dm^{-3} S4d, and 10 mmol dm^{-3} S4e were used. The resulting H_2O_2 concentrations for all 2-propanol (IPA) concentrations are also plotted as a function of sonication time S4f.

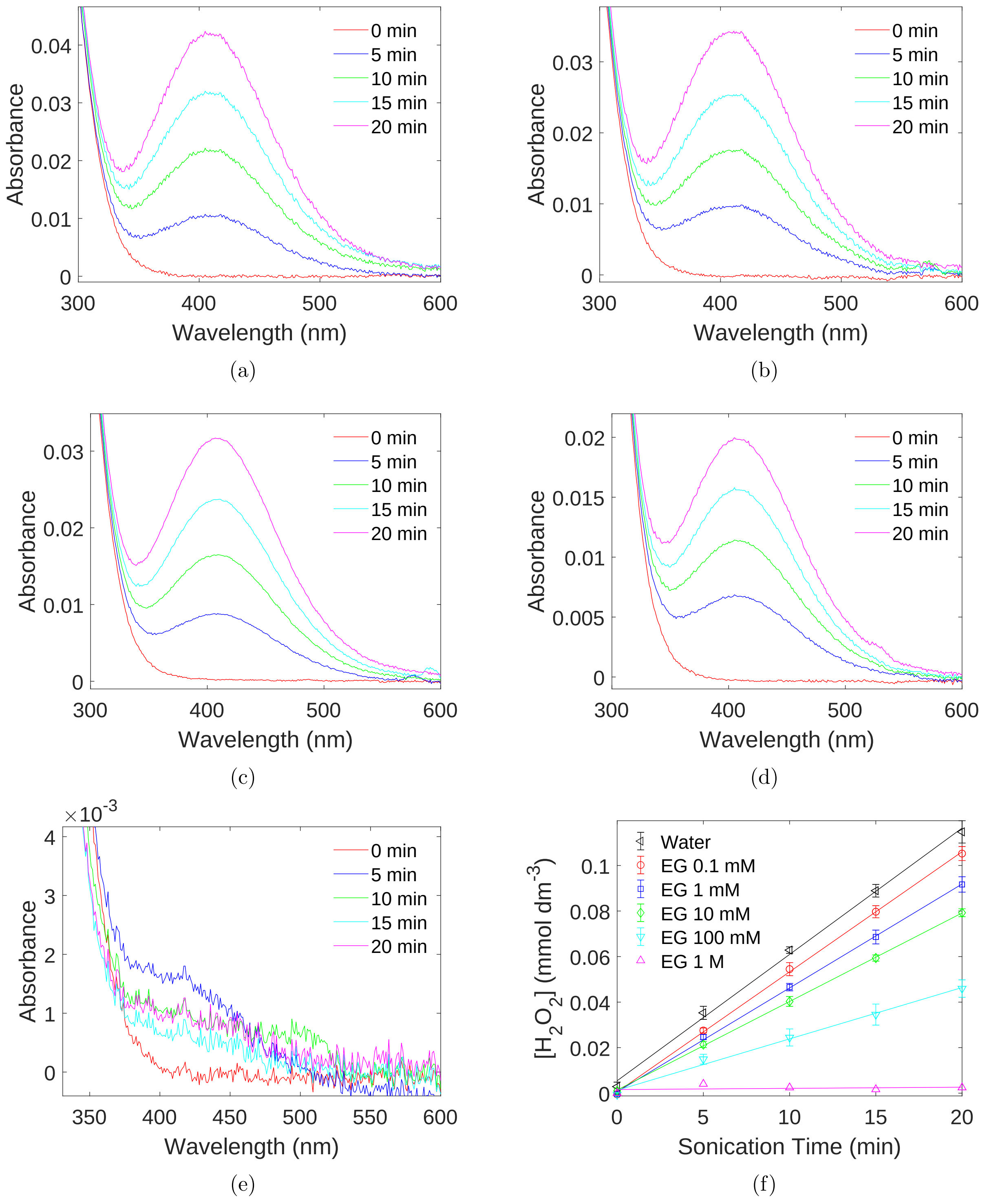

Figure S5: Absorbance spectra of titanium hydrogen peroxide complexes at different sonication times acquired using ethylene glycol as the radical scavenger. Initial ethylene glycol concentrations of $0.1\,\mathrm{mmol\,dm^{-3}}$ S5a, $1.0\,\mathrm{mmol\,dm^{-3}}$ S5b, $10\,\mathrm{mmol\,dm^{-3}}$ S5c, $100\,\mathrm{mmol\,dm^{-3}}$ S5d, and $1.0\,\mathrm{mol\,dm^{-3}}$ S5e were used. The resulting H_2O_2 concentrations for all ethylene glycol (EG) concentrations are also plotted as a function of sonication time S5f.

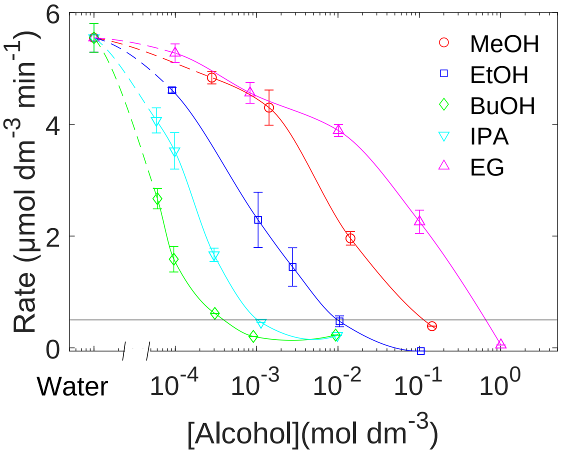

Figure S6: Rate of H_2O_2 formation as a function of alcohol concentration. Methanol (MeOH) (○), ethanol (EtOH) (□), 1-butanol (BuOH) (◇), 2-propanol (IPA) (▽), and ethylene glycol (EG) (△) were used as radical scavengers. Solid lines are drawn with spline interpolation to guide the eye. The horizontal line represents the lower detection limit of H_2O_2.

Silver Nanoparticle Absorbance Spectra

Absorbance spectra showing the development of the Ag plasmon peak for different sonication times along with their corresponding concentration profiles are given in Figure S7, S8, S9, S10, and S11 for methanol, ethanol, 1-butanol, 2-propanol, and ethylene glycol, respectively. The rates of Ag-nanoparticle formation for different alcohols and alcohol concentrations are shown in Figure S12. The absorbance values are not corrected for shifts in the Ag LSPR peak.

We would also like to briefly address the validity of the method used to quantify the rate of Ag-nanoparticle formation. Considering the short sonication time (20 min), the relatively high initial Ag(I) concentration (1 mmol dm^{-3}), the one-electron transfer from Ag(I) to Ag(0), and the fact that formation of primary (and secondary) radicals is linearly dependent on time, we also expect the Ag-nanoparticle formation to behave linearly as well within this short time frame. From the concentration profiles in the supporting information (Figure S7-S11), we observe that the Ag-nanoparticle formation is indeed linear. All concentration profiles appear to be quite linear except for the optimum concentrations for ethylene glycol (where pyrolytic decomposition is significant). Interference from the asymmetric or overlapping peaks therefore appear to not interfere much with the analysis of the results. Certainly not enough to change the clearly different trends observed in Figure S12.

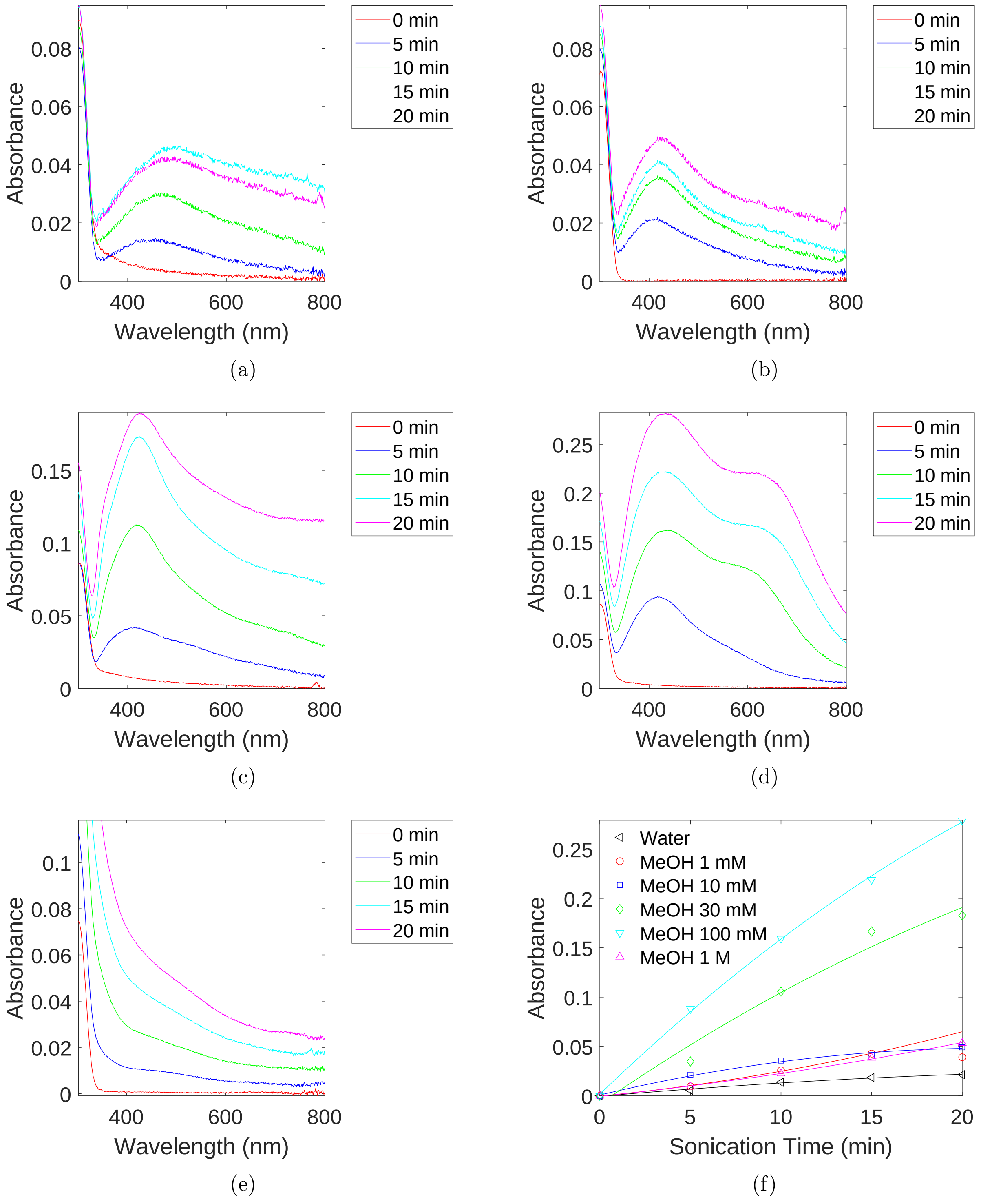

Figure S7: Absorbance spectra of Ag-nanoparticles at different sonication times acquired using methanol as the radical scavenger. Initial methanol concentrations of $1.0\,\mathrm{mmol\,dm^{-3}}$ S7a, $10\,\mathrm{mmol\,dm^{-3}}$ S7b, $30\,\mathrm{mmol\,dm^{-3}}$ S7c, $100\,\mathrm{mmol\,dm^{-3}}$ S7d, and $1.0\,\mathrm{mol\,dm^{-3}}$ S7e were used. The absorbance for all methanol (MeOH) concentrations are also plotted as a function of sonication time S7f.

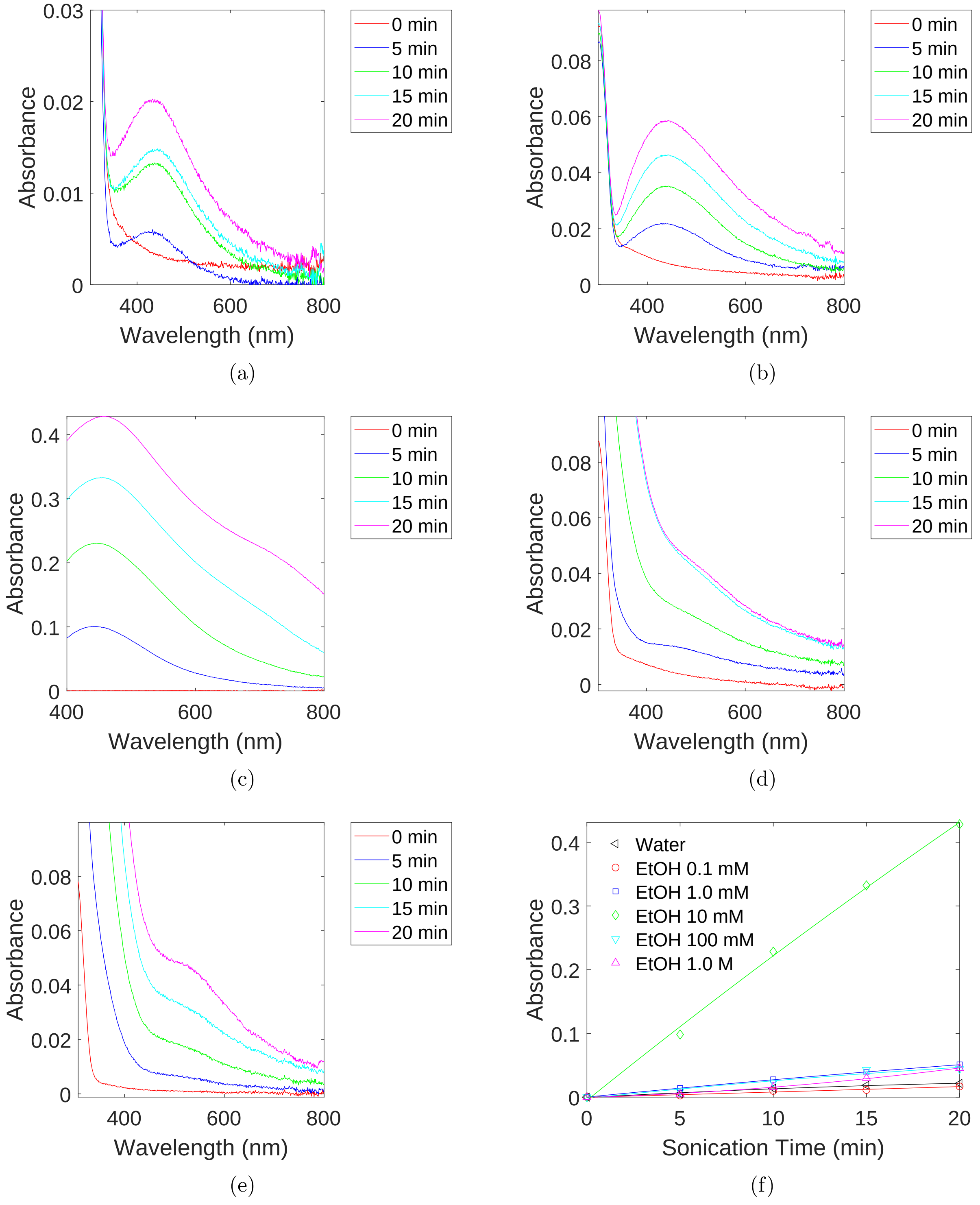

Figure S8: Absorbance spectra of Ag-nanoparticles at different sonication times acquired using ethanol as the radical scavenger. Initial ethanol concentrations of 0.1 mmol dm^{-3} S8a, 1.0 mmol dm^{-3} S8b, 10 mmol dm^{-3} S8c, 100 mmol dm^{-3} S8d, and 1.0 mol dm^{-3} S8e were used. The absorbance for all ethanol (EtOH) concentrations are also plotted as a function of sonication time S8f.

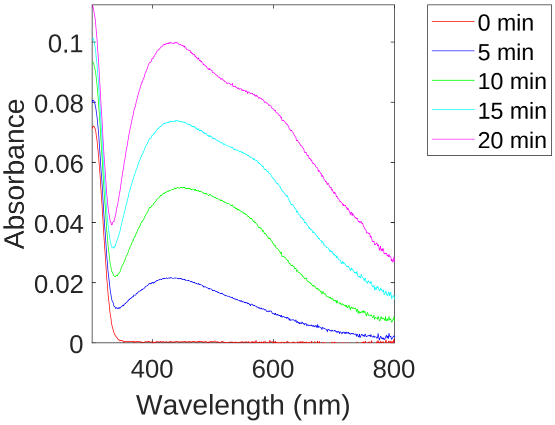

(a)

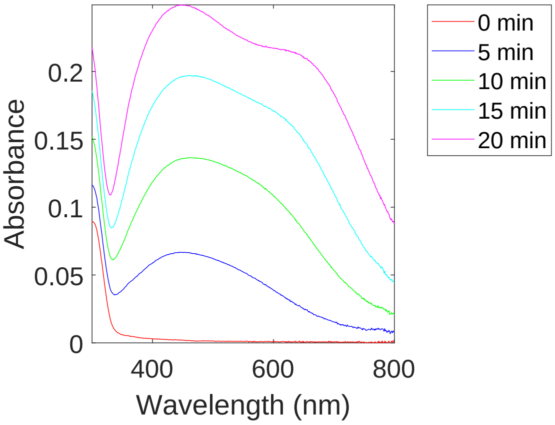

(b)

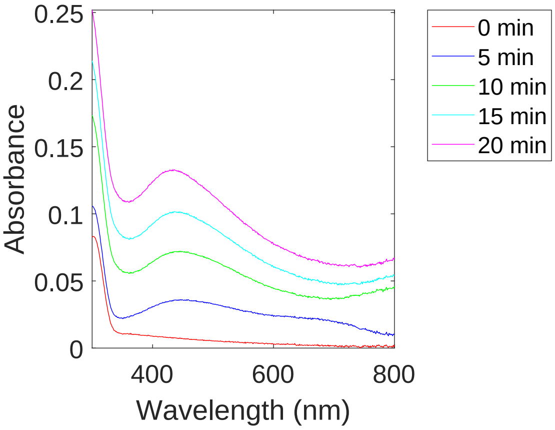

(c)

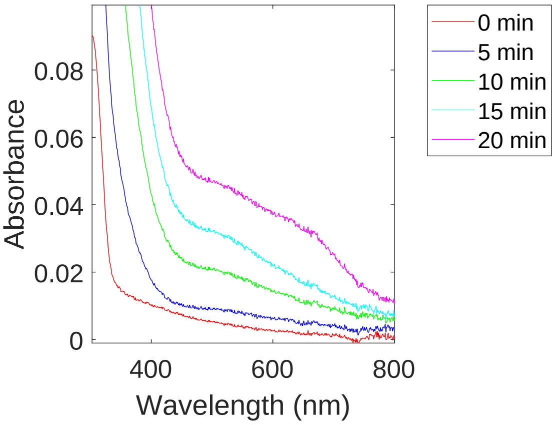

(d)

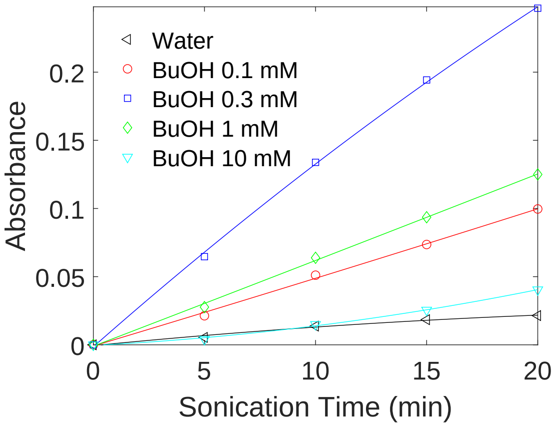

(e)

Figure S9: Absorbance spectra of Ag-nanoparticles at different sonication times acquired using 1-butanol as the radical scavenger. Initial 1-butanol concentrations of $0.1\,\mathrm{mmol\,dm^{-3}}$ S9a, $0.3\,\mathrm{mmol\,dm^{-3}}$ S9b, $1.0\,\mathrm{mmol\,dm^{-3}}$ S9c, and $10\,\mathrm{mmol\,dm^{-3}}$ S9d were used. The absorbance for all 1-butanol (BuOH) concentrations are also plotted as a function of sonication time S9e.

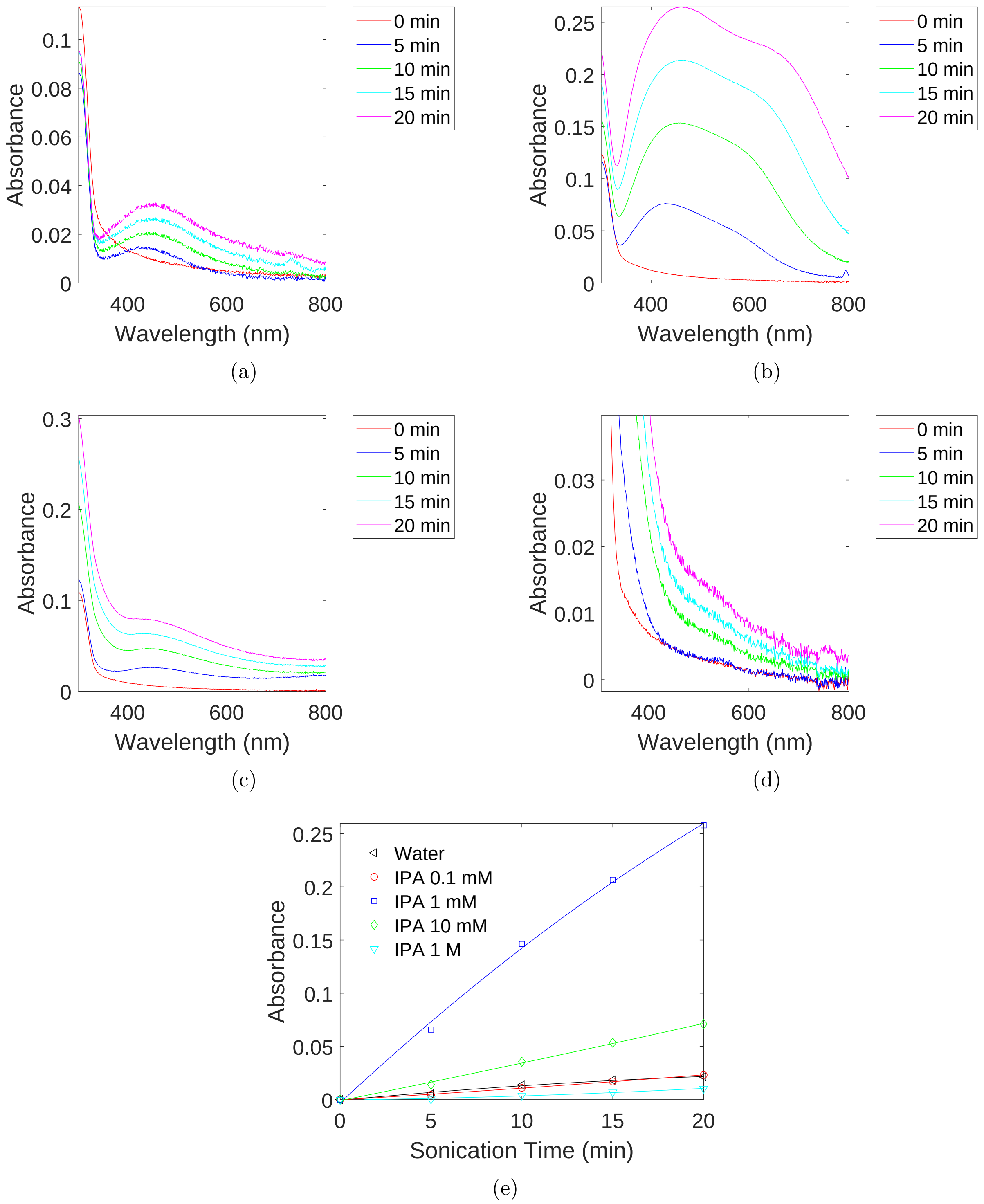

Figure S10: Absorbance spectra of Ag-nanoparticles at different sonication times acquired using 2-propanol as the radical scavenger. 2-propanol concentrations of 0.1 mmol dm^{-3} S10a, 1.0 mmol dm^{-3} S10b, 10 mmol dm^{-3} S10c, and 1.0 mol dm^{-3} S10d were used. The absorbance for all 2-propanol (IPA) concentrations are also plotted as a function of sonication time S10e.

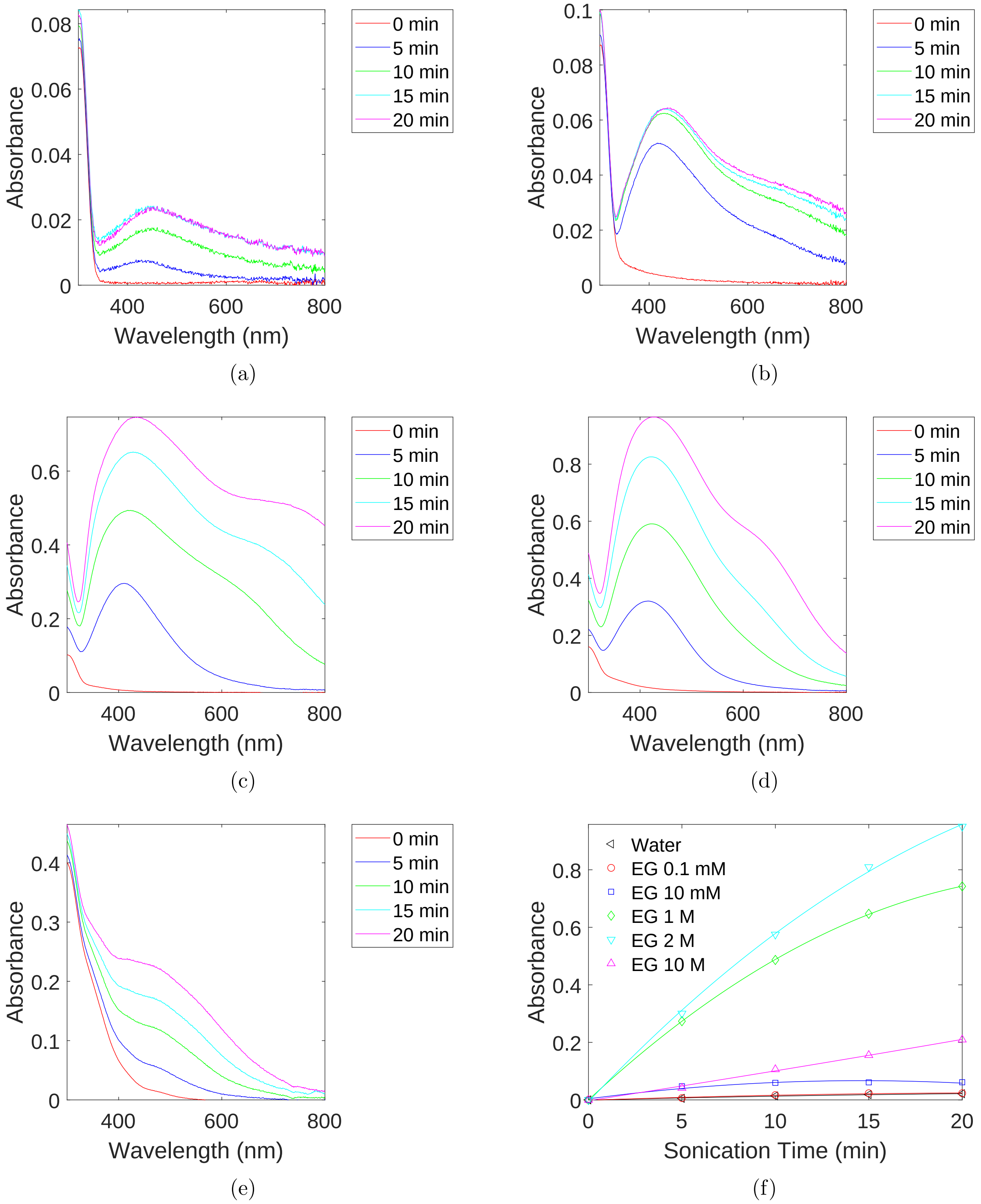

Figure S11: Absorbance spectra of Ag-nanoparticles at different sonication times acquired using ethylene glycol as the radical scavenger. Initial ethylene glycol concentrations of 0.1 mmol dm^{-3} S11a, 10 mmol dm^{-3} S11b, 1.0 mol dm^{-3} S11c, 2.0 mol dm^{-3} S11d, and 10 mol dm^{-3} S11e were used. The absorbance for all ethylene glycol (EG) concentrations are also plotted as a function of sonication time S11f.

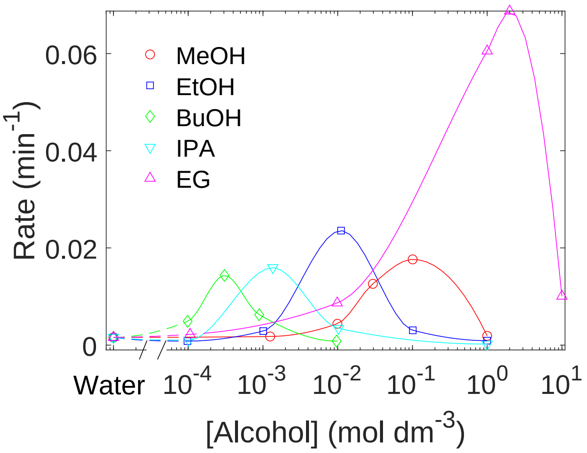

Figure S12: Rate of Ag-nanoparticle formation as a function of alcohol concentration. Methanol (MeOH) (○), ethanol (EtOH) (□), 1-butanol (BuOH) (◇), 2-propanol (IPA) (▽), and ethylene glycol (EG) (△) were used as radical scavengers. The rates of Ag formation are extracted from the slopes of the absorbance profiles and are not corrected for shifts in the localized surface plasmon resonance peaks. Solid lines are drawn with spline interpolation to guide the eye.

Scavenging Efficiency of Nitrate

Concentration profiles of H_2O_2 in water and in 10 mmol dm^{-3} $NaNO_3$ are shown in Figure S13. Both concentration profiles overlap completely showing that the nitrate ions which are present during the Ag synthesis does not contribute to any scavenging of primary radicals.

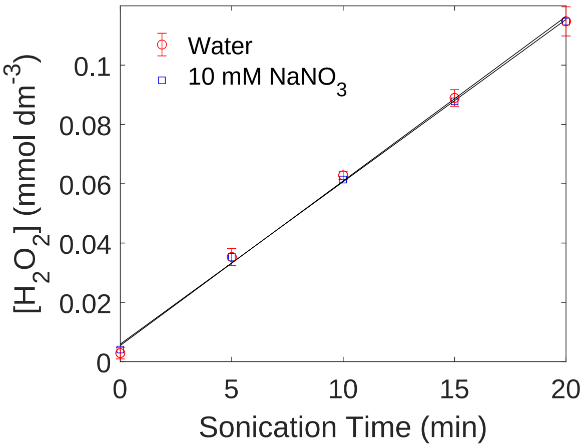

Figure S13: Concentration of H_2O_2 as a function of sonication time in water (○) and in 10 mmol dm^{-3} $NaNO_3$ (□). The solid lines are linear fits to the datapoints.

Electron Microscopy

SEM micrographs of Ag-nanoparticles synthesized with ethylene glycol (2 mmol dm^{-3}) as the scavenger is shown in Figure S14a. Micrographs were acquired with a Hitachi High-Tech SU9000 scanning (tunneling) electron microscope using an acceleration voltage of 20 kV and a beam current of 10.5 µA. An energy-dispersive X-ray spectroscopy (EDS) map of Ag for the same particle is shown in Figure

S14b. EDS was performed with an Oxford Ultim Extreme EDX-system. Both single nanoparticles and agglomerates were observed.

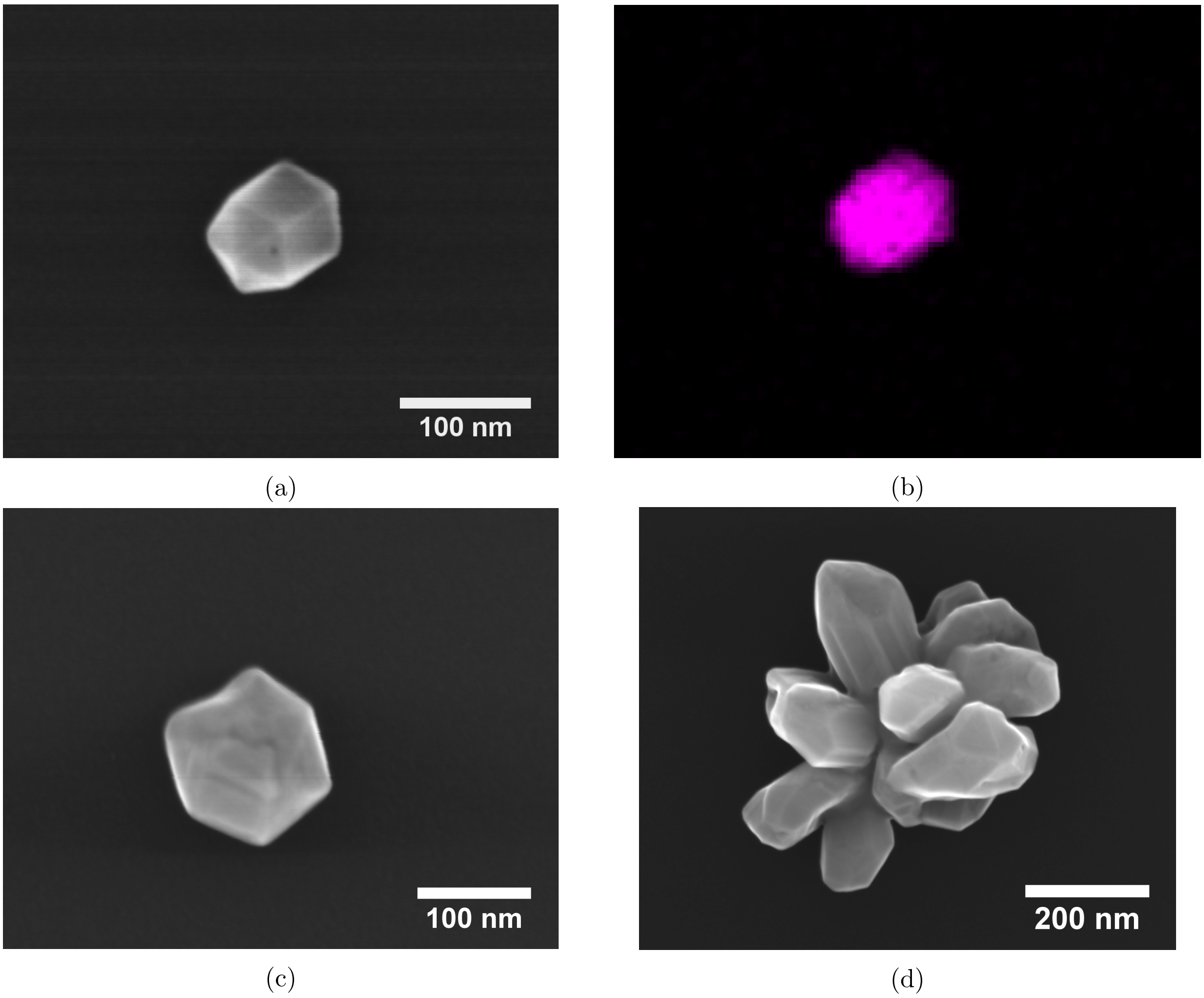

Figure S14: Secondary electron image (S14a) and the corresponding EDS map of Ag (S14b) synthesized with ethylene glycol as the radical scavenger. Additional images of a single nanoparticle (S14c) and an agglomerated particle (S14d) are also provided.

Platinum(II)/Platinum(IV) Absorbance Spectra

Absorbance spectra and the corresponding Pt(II) concentration profiles for all methanol concentrations are shown in Figure S15. The rate of Pt(II) formation at different methanol concentrations are given in Figure S16.

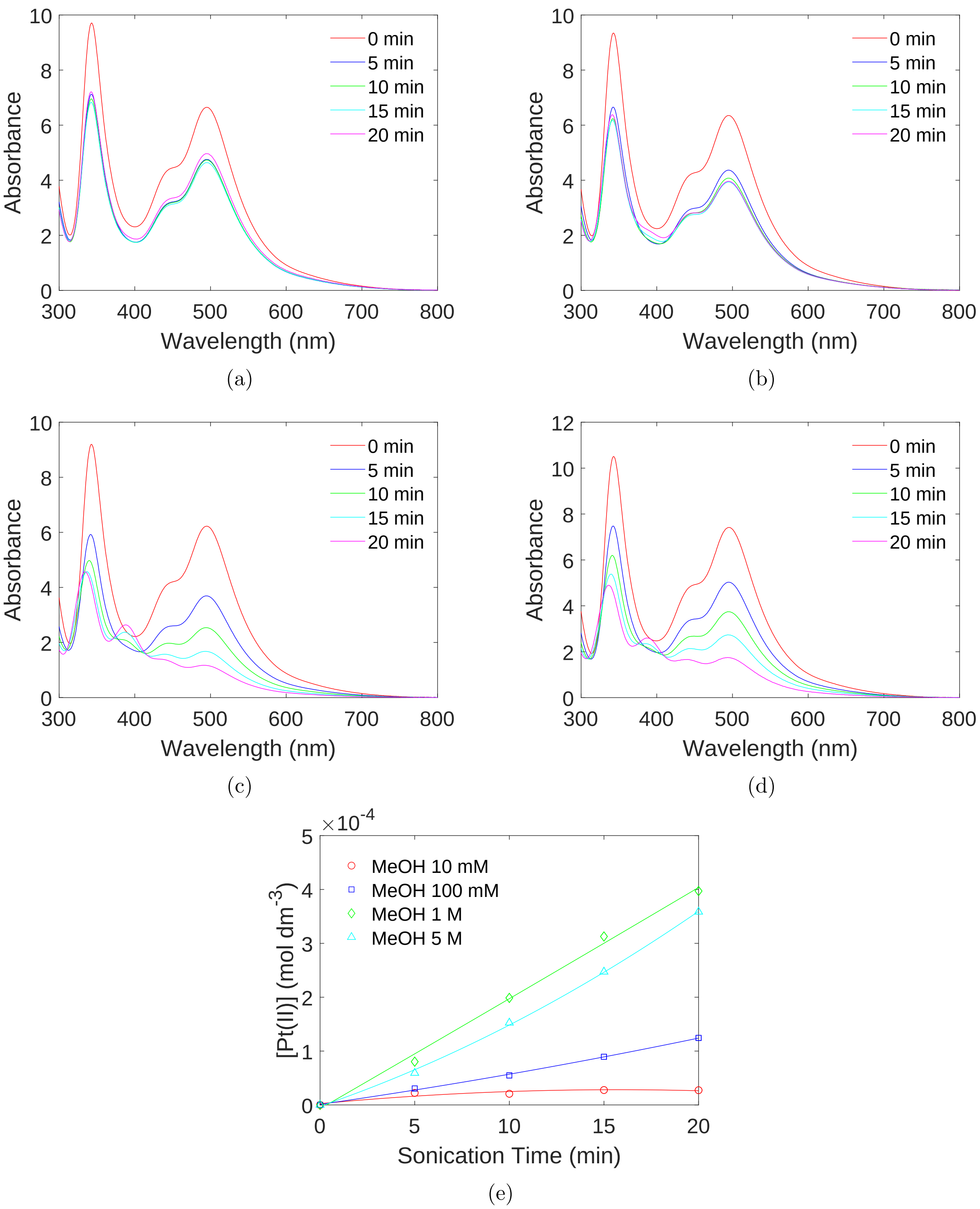

Figure S15: Absorbance spectra of Pt(IV)/Pt(II)-iodide complexes at different sonication times acquired using methanol as the radical scavenger. Initial methanol concentrations of 10 mmol dm^{-3} S15a, 100 mmol dm^{-3} S15b, 1.0 mol dm^{-3} S15c, and 5.0 mol dm^{-3} S15d were used. The Pt(II) concentrations for all methanol (MeOH) concentrations are also plotted as a function of sonication time S15e.

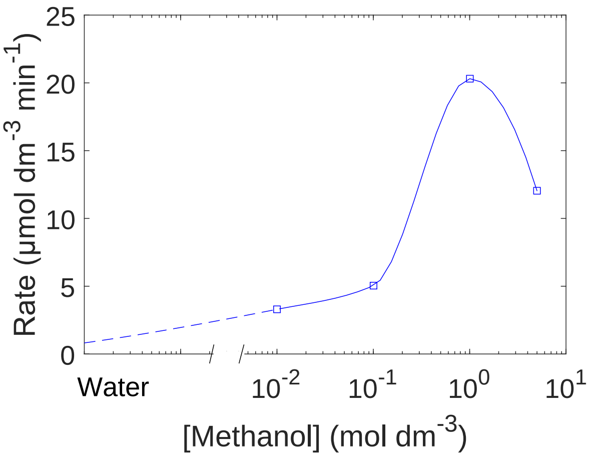

Figure S16: Rate of Pt(II) formation as a function of methanol concentration. The solid lines are drawn with spline interpolation to guide the eye.

Absorbance Spectra at 30 W and 760 kHz

Absorbance spectra of H_2O_2 for different sonication times along with their corresponding concentration profiles are given in Figure S17. The measurements were conducted using an ultrasonic frequency of 346 kHz and an electrical power of 30 W with methanol as the radical scavenger.

Absorbance spectra of H_2O_2 for different sonication times along with their corresponding concentration profiles are given in Figure S18. The measurements were conducted using an ultrasonic frequency of 760 kHz and an electrical power of 50 W with methanol as the radical scavenger.

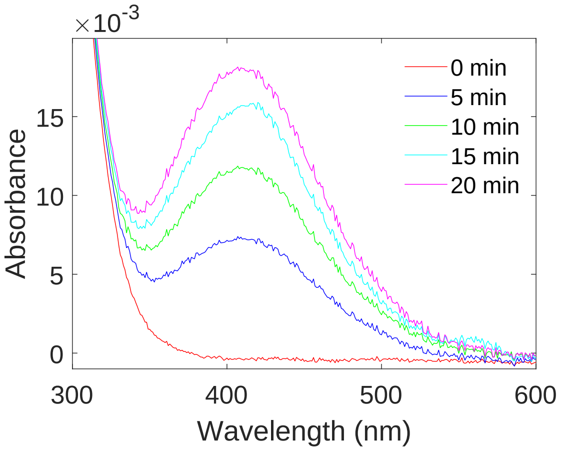

(a)

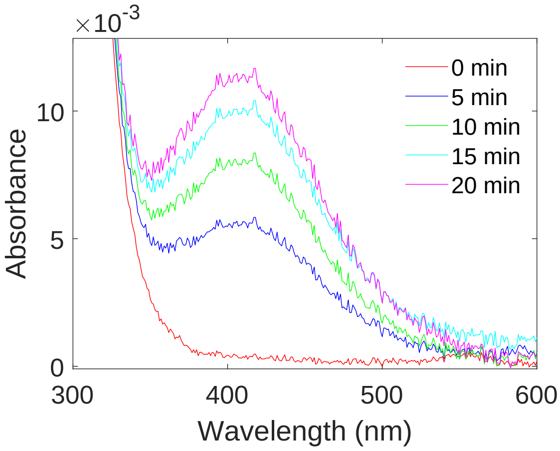

(b)

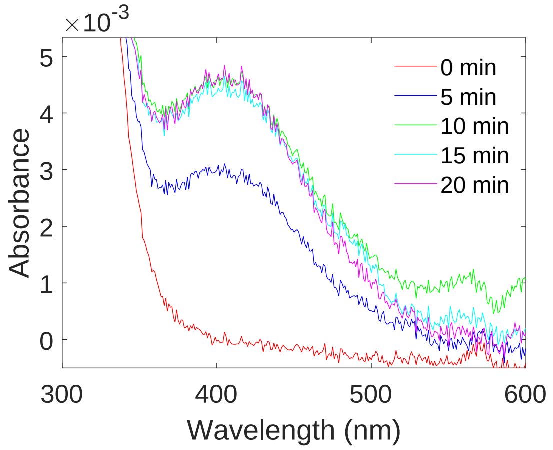

(c)

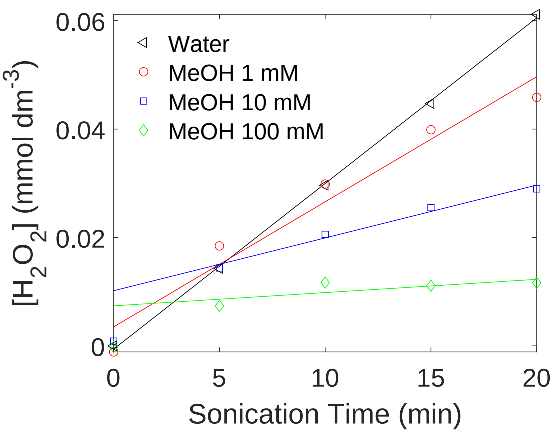

(d)

Figure S17: Absorbance spectra of H_2O_2 at different sonication times acquired with an electrical power of 30 W, an ultrasonic frequency of 346 kHz, and methanol as the radical scavenger. Initial methanol concentrations of 1.0 mmol dm^{-3} S17a, 10 mmol dm^{-3} S17b, and 100 mmol dm^{-3} S17c were used. The resulting H_2O_2 concentrations for all methanol (MeOH) concentrations are also plotted as a function of sonication time S17d.

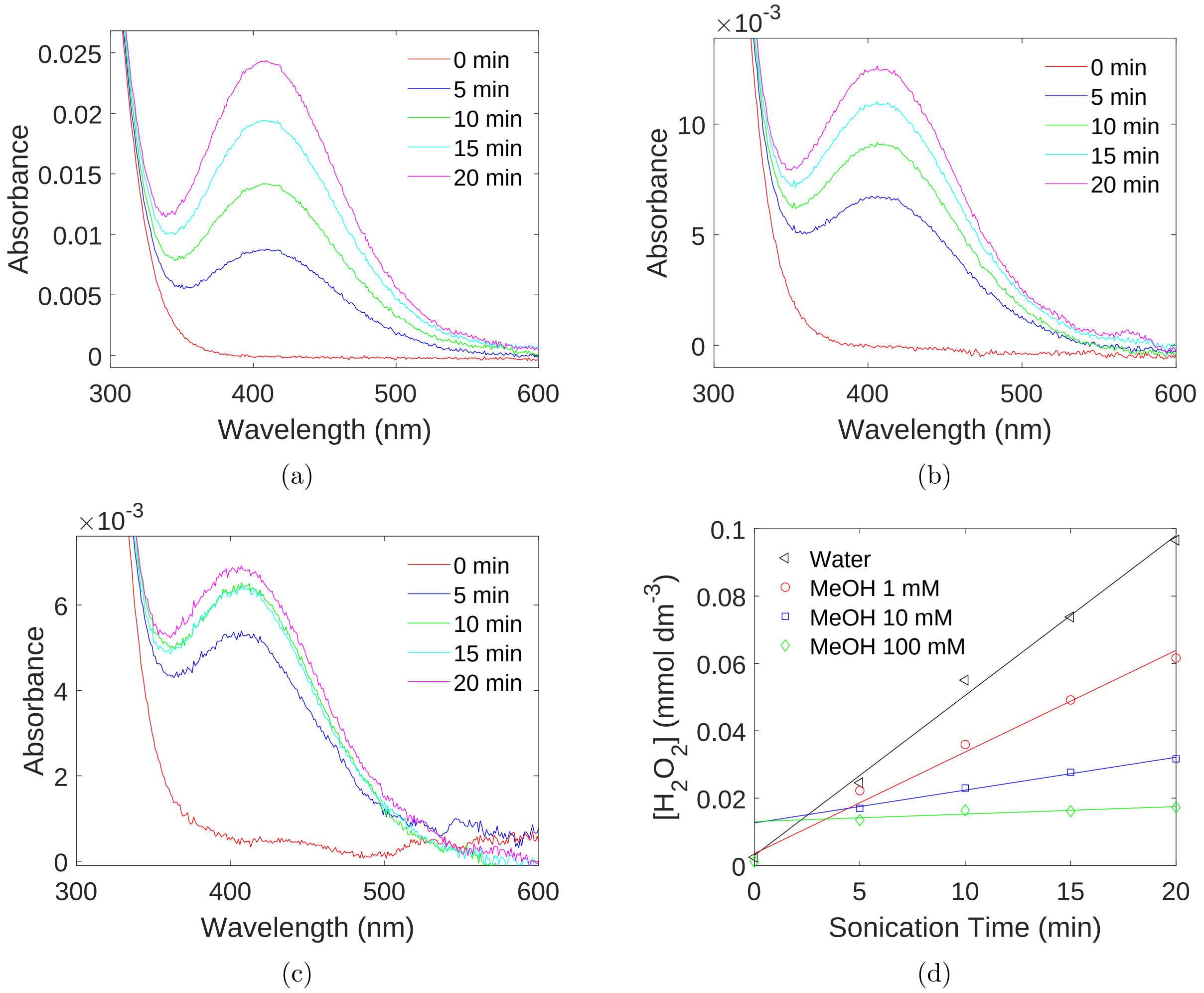

Figure S18: Absorbance spectra of H_2O_2 at different sonication times acquired with an ultrasonic frequency of 760 kHz, an electrical power of 50 W, and methanol as the radical scavenger. Initial methanol concentrations of $1.0\,\text{mmol}\,\text{dm}^{-3}$ S18a, $10\,\text{mmol}\,\text{dm}^{-3}$ S18b, and $100\,\text{mmol}\,\text{dm}^{-3}$ S18c were used. The resulting H_2O_2 concentrations for all methanol (MeOH) concentrations are also plotted as a function of sonication time S18d.

Localized Surface Plasmon Resonance Peak Correction

The calibration curve used to correct the absorbance at the LSPR peak for Ag nanoparticles is shown in Figure S19.

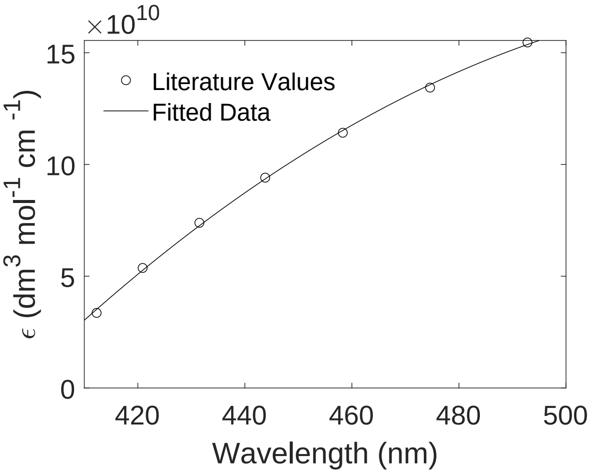

Figure S19: Molar extinction coefficient of Ag-nanoparticles as a function of the localized surface plasmon resonance peak in the absorbance spectrum. Experimental values (○) are obtained from Paramelle et al. [33]. The data were fitted to a 2. order polynomial and is plotted as a solid line.
